# The role of ataluren in the treatment of ambulatory and non-ambulatory children with nonsense mutation duchenne muscular dystrophy - a consensus derived using a modified Delphi methodology in Eastern Europe, Greece, Israel and Sweden

**DOI:** 10.1186/s12883-024-03570-x

**Published:** 2024-02-21

**Authors:** Tanja Golli, Lenka Juříková, Thomas Sejersen, Craig Dixon

**Affiliations:** 1 Department of Child, Adolescent and Developmental Neurology, Ljubljana University Medical Centre, Ljubljana, Slovenia; 2 Department of Pediatric Neurology, Faculty of Medicine, University Hospital Brno, Masaryk University in Brno, Brno, Moravia Czech Republic; 3 Department of Women’s and Children’s Health, Karolinska Institute, Stockholm, Sweden; 4 MASS Team, Suite 99, 95 Mortimer Street, London, W1W 7GB UK

**Keywords:** Ataluren, Consensus, Disease progression, Duchenne muscular dystrophy, Dystrophin, Non-ambulatory

## Abstract

**Background:**

This paper details the results of an evaluation of the level of consensus amongst clinicians on the use of ataluren in both ambulatory and non-ambulatory patients with nonsense mutation Duchenne muscular dystrophy (nmDMD). The consensus was derived using a modified Delphi methodology that involved an exploration phase and then an evaluation phase.

**Methods:**

The exploration phase involved 90-minute virtual 1:1 interviews of 12 paediatric neurologists who cared for 30–120 DMD patients each and had patient contact every one or two weeks. The respondents managed one to ten nmDMD patients taking ataluren. The Discussion Guide for the interviews can be viewed as Appendix A. Following the exploration phase interviews, the interview transcripts were analysed by an independent party to identify common themes, views and opinions and developed 43 draft statements that the Steering Group (authors) reviewed, refined and endorsed a final list of 42 statements. Details of the recruitment of participants for the exploration and evaluation phases can be found under the Methods section.

**Results:**

A consensus was agreed (> 66% of respondents agreeing) for 41 of the 42 statements using results from a consensus survey of healthcare professionals (*n* = 20) experienced in the treatment of nmDMD.

**Conclusions:**

The statements with a high consensus suggest that treatment with ataluren should be initiated as soon as possible to delay disease progression and allow patients to remain ambulatory for as long as possible. Ataluren is indicated for the treatment of Duchenne muscular dystrophy that results from a nonsense mutation in the dystrophin gene, in ambulatory patients aged 2 years and older (see Summary of Product Characteristics for each country)

**Supplementary Information:**

The online version contains supplementary material available at 10.1186/s12883-024-03570-x.

## Background

Duchenne muscular dystrophy (DMD) is a genetic disease that causes muscle weakness and wasting. Children born with DMD have a mutation in the dystrophin gene [1]. The dystrophin gene is made up of 79 exons coding for a protein of 3,685 amino acid residues [2]. Dystrophin is a cohesive protein, linking actin filaments to other support proteins that reside on the inside surface of each muscle fibres’ plasma membrane (sarcolemma) [1, 3]. Without dystrophin, muscles get damaged more easily, and muscle strength and function is weakened. The dystrophin gene is located on the X chromosome, hence DMD almost always affects boys, and they tend to be diagnosed before the age of five [1, 3].

The treatment of DMD aims to improve quality of life, delay disease progression and increase life expectancy; it requires a multidisciplinary approach. Despite major therapeutic advances over the past 30 years, there is still no cure for DMD [4–6].

Corticosteroids remain the main drug treatment for DMD [4–6], and their use can prolong ambulation and improve everyday functionality [7, 8]. Longer term, they can reduce the need for scoliosis surgery, enhance lung function, and help maintain cardiac function but there are severe issues with side effects such as weight gain, impaired linear growth, mood disturbance, hypertension and immunosuppression, to name just a few [6]. There is, therefore, an unmet need for treatments that can be used in DMD patients longer term into the non-ambulatory phase which have a positive impact on disease progression with an improved safety profile compared with corticosteroids. Ataluren is indicated for the treatment of Duchenne muscular dystrophy that results from a nonsense mutation in the dystrophin gene, in ambulatory patients aged 2 years and older in the European Member States and Iceland, Liechtenstein, Norway, Great Britain, Northern Ireland, Kazakhstan, Israel, Republic of Korea, Belarus, Russia, and Brazil, and aged 5 years and older in Chile, the Kingdom of Saudi Arabia, and Ukraine (under special state registration). In Brazil, the indication is specific to male paediatric patients. The presence of a nonsense mutation in the dystrophin gene should be determined by genetic testing (Translarna Summary of Product Characteristics (SmPC) for respective countries) [9]. Corticosteroids, the standard of care, are not disease-specific and used off-license, to treat symptoms only whereas ataluren is a disease-specific and disease-modifying therapy, mostly used in addition to corticosteroids. Ataluren interacts with ribosomes, enabling it to neglect premature nonsense stop signals on mRNA, thereby allowing the cell to produce a full-length, functional dystrophin protein.

Since its approval, several years of real-world clinical experience have increased our knowledge of the impact of ataluren treatment in patients with nmDMD in terms of delay in loss of muscle function in limbs/ability to ambulate [10], a delay in progression in patients who are non-ambulatory [10, 11], that includes maintenance of cardiac function [12] and maintenance of pulmonary function [11]. These benefits of ataluren in clinical practice were seen in patients who also received the usual standard of care (including corticosteroids) [10, 11]. We wanted to gather real-world data on the use of ataluren in the treatment of ambulatory and non-ambulatory DMD patients in order to establish a consensus on its use. The objectives of this activity were to gain such a consensus, using a modified Delphi methodology, on clinicians’ own experience of the use of ataluren in patients with nmDMD and their opinions on the value of ataluren in ambulatory and non-ambulatory patients with nmDMD. The overarching aim of this consensus is to improve clinical practice and enhance patient’s lives.

## Methods

PTC therapeutics international initiated and fully supported this project and commissioned the MASS Team, a healthcare consultancy, to independently facilitate the project and analyse the responses to the consensus statements, in line with this modified Delphi methodology. The Delphi method (modified in this case) is a well-established, systematic approach to answering research questions through the identification of a consensus view across subject experts [13, 14]. PTC therapeutic international, wanted to support a study that examined nmDMD treatment with ataluren in the CEE region, as many studies are already focused on western European countries, and therefore determined the countries to be included in the study.

An exploration phase was conducted to identify common themes and opinions. To identify possible advisors for the exploration phase, we searched clinicaltrials.gov using the terms ‘Duchenne Muscular Dystrophy’ and ‘Ataluren’ which returned 16 clinical studies. These studies were examined for trial centres within the countries included in the study scope, this identified study centres in all countries with the exceptions of Croatia. To identify centres with an expertise in DMD in the remaining country authors from the TREAT-NMD DMD Global Database were examined and potential respondents identified.

The exploration phase involved one-to-one interviews of approximately 90 min, conducted by the MASS Team using Microsoft teams, with 12 clinicians from nine countries, as listed in Table 1. The 12 clinicians interviewed were paediatric neurologists, cared for 30–120 DMD patients each and had patient contact every one or two weeks. The respondents managed one to ten nmDMD patients taking ataluren. The Discussion Guide for the interviews can be viewed as Appendix A.

**Table 1:** Clinicians who completed the exploration phase

Country	Number of clinicians
Bulgaria	2
Croatia	1
Czech Republic	2
Greece	1
Hungary	2
Israel	1
Romania	1
Slovenia	1
Sweden	1
Total countries: 9	Total interviewed: 12

The MASS team manually analysed the transcripts from the interviews using an inductive coding approach, a single reviewer conducted the analysis, this was then independently reviewed by another member of the MASS team, who also reviewed transcripts, before the draft statements were approved by both. 43 draft statements were developed and the following broad themes emerged:

- Progression of nmDMD.
- Loss of ambulation.
- Scoliosis.
- Upper limb function.
- Pulmonary function.
- Cardiac function.
- Treatment with ataluren.
- Duration of treatment with ataluren.

Three physicians who took part in the Exploration phase interviews were then invited to form a Steering Group to review, refine and ultimately approve the statements and inform the subsequent process for achieving consensus with a wider group. The Steering Group met for three hours, via Microsoft teams, and ultimately approved 42 statements for use in a wider evaluation phase. It was determined that the evaluation phase would be conducted using an online questionnaire (on the platform surveymonkey.com) which would invite respondents to indicate their level of individual agreement with each statement using a four-point Likert scale, which allowed respondents to record levels of agreement with each statement and suggest changes, in a free text field, as appropriate. The four-point Likert scale forces either agreement or disagreement with each statement (strongly disagree, disagree, agree, or strongly agree). The Steering Group defined the threshold for consensus as ≥ 66% of respondents selecting ‘agree’ or ‘strongly agree’ for each statement, with the consensus defined as ‘high’ if ≥ 66% of respondents agreed and ‘very high’ if ≥ 90% of respondents agreed. The four-point Likert scale and similar thresholds for consensus have been utilised in other similar studies [15–18].

The Steering Group agreed if consensus was not achieved for greater than 66% of the statements in the first evaluation phase then the Delphi process would proceed, and a second evaluation phase would take place. In the event that a large proportion of statements were considered contentious in the first evaluation phase then an additional steering group meeting would be required to review and further develop the statements through an iterative process. Subsequently, if consensus were not achieved for greater than 66% of the statements after the second evaluation phase, then the decision would be taken not to proceed to a third evaluation phase and proceed straight to the data analysis. If consensus were achieved for greater than 66% of the statements in the first evaluation phase a second evaluation phase would not be deemed necessary, and the Steering Group would meet once again to review and discuss the results.

To identify clinicians with experience treating nmDMD patients and clinical experience with ataluren the Steering Group recommended that potential respondents for the evaluation phase were identified and invited to participate by TREAT-NMD. TREAT-NMD supported recruitment of prescribing clinicians in Europe from Bulgaria, Croatia, Czech Republic, Hungary and Slovenia through their participation in the TREAT-NMD DMD registry. A member of the Steering Group identified clinicians in Slovakia, whilst the MASS Team contacted clinicians involved in the exploration phase in Greece, Israel and Romania and invited them to extend the invitation to their peers. Potential respondents were invited to participate via an email which contained a link to the online questionnaire. Participants in the evaluation phase(s) would be paid a nominal honorarium of £75.00 for the time to complete the questionnaire. The Steering Group members were excluded from inclusion in the evaluation phase.

## Results

The first evaluation phase resulted in online questionnaires being completed by 20 respondents from eight countries. 75% were paediatric neurologists, 15% consultant paediatric neurologists*, 5% **neurologists** and 5% paediatricians.

A Consensus was achieved for 41 of the 42 (> 97%) statements so, inline with the agreed methodology, there was no requirement to complete a second evaluation phase.

The results of the evaluation phase are detailed in Appendix C. The raw data can be viewed in Appendix D.

### Progression of nmDMD

The loss of motor function is both clinically important and meaningful to patients and their families [11] because the progressive loss of each function is usually irreversible [11, 19]. 

Four statements relating to the progression of nmDMD were included in the evaluation phase and there was a ‘high’ and ‘very high’ consensus in all proposed statements. (See Table 2)

**Table 2:** Consensus for statements on the progression of nmDMD indicate that respondents agreed that the decline of muscle function of the lower limbs, the first to decline, can lead to loss of ambulation and that a decline in cardiac and pulmonary function are two of the major causes of death

Theme	Statement	Overall Agreement	Consensus
Progression of nmDMD	The speed of progression of nmDMD is variable and individual to the patient	85.0%	High
	The earlier nmDMD patients are diagnosed and treatment initiated, the greater the delay in muscle decline	95.0%	Very high
	Proximal lower limb muscles are amongst the first to decline in nmDMD leading to loss of ambulation	100.0%	Very high
	Decline in cardiac and pulmonary function are two of the major causes of death in nmDMD	100.0%	Very high

The threshold for consensus defined as > 66%, with consensus being defined as high at > 66% and very high at > 90% of respondents selecting agree or strongly agree

Results indicated that there is a very high consensus that a greater delay in muscle decline can be achieved if nmDMD patients are diagnosed earlier and treatment initiated as soon as possible.

### Loss of ambulation

Preservation of motor function impacts a patient’s autonomy and quality of life by postponing the loss of basic daily functions, such as climbing and descending stairs, and walking short distances independently [20]. Furthermore, the time of loss of one function predicts the onset of subsequent disease milestones indicative of disease progression [21]. 

There was a consensus on almost all of the seven statements included in the evaluation phase on the loss of ambulation in nmDMD patients and these can be viewed in Table 3. However, there was no consensus (65% agreed) on the statement ‘Ataluren (in addition to standard of care) is expected to result in the same treatment effect in each surviving muscle fibre irrespective of the nmDMD patients’ ambulatory status’. This was the only statement of the entire survey that did not achieve a consensus.

**Table 3:** Results indicating that there was a high consensus that ambulation in patients should be preserved for as long as possible

Theme	Statement	Overall Agreement	Consensus
Loss of ambulation	Ataluren (in addition to standard of care) significantly delays the loss of ambulation in patients with nmDMD	85.0%	High
	Ataluren (in addition to standard of care) is expected to result in the same treatment effect in each surviving muscle fibre irrespective of the nmDMD patients’ ambulatory status	65.0%	None
	If a nmDMD patient receiving ataluren loses ambulation, they should continue treatment with ataluren	95.0%	Very high
	There is life beyond loss of ambulation. There are still lots of important functions of the muscles such as being able to use the hands and arms, fine motor skills and respiratory muscles – all these functions should be maintained for as long as possible	100.0%	Very high
	Delaying the loss of ambulation in patients with nmDMD may reduce the development of scoliosis	90.0%	High
	Delaying the loss of ambulation in patients with nmDMD delays the decline of respiratory function	100.0%	Very high
	Delaying the loss of ambulation in patients with nmDMD is related to the decline of upper limb function	75.0%	High

The threshold for consensus defined as > 66%, with consensus being defined as high at > 66% and very high at > 90% of respondents selecting agree or strongly agree

Clinicians, however, did agree that loss of ambulation should be preserved for as long as possible in patients with nmDMD as this can have a positive impact on the development of other muscle function decline. They also agreed that ataluren, in addition to standard of care, significantly delays the loss of ambulation in patients with nmDMD.

### Scoliosis and upper limb function

Patients with DMD often develop scoliosis that progresses rapidly after loss of ambulation [22]. Management of scoliosis is crucial because it affects both life expectancy and quality of life in patients with DMD [22]. A number of statements included in the evaluation phase considered scoliosis.

Results indicated that 100% of clinicians agreed that the development of scoliosis has a detrimental impact on patients’ pulmonary function and 85% agreed that patients treated with ataluren, in addition to standard of care, are less likely to develop scoliosis (Table 4).

**Table 4:** Evaluation phase results indicating that treatment of nmDMD patients with ataluren, in addition to standard of care, can delay both scoliosis and the decline in patients’ upper limb function, regardless of mobility status

Theme	Statement	Overall Agreement	Consensus
Scoliosis	Development of scoliosis has a detrimental impact on patients’ pulmonary function	100.0%	Very high
	nmDMD patients treated with ataluren (in addition to standard of care) are less likely to develop scoliosis	85.0%	High
	Non-ambulatory nmDMD patients are less likely to develop scoliosis if they continue treatment with ataluren (in addition to standard of care) after loss of ambulation	75.0%	High
Upper limb function	Delaying the decline of muscle function in patients’ upper limbs helps to maintain independence	100.0%	Very high
	Delaying the decline in fine motor skills also enables patients in wheelchairs to continue to be as independent as possible	100.0%	Very high
	Delaying the decline of upper limb strength enables non-ambulatory patients to transfer from their wheelchair to the toilet, maintain intimate hygiene, retain independence and protect their quality of life	100.0%	Very high
	Decline of upper limb function has a major impact on patients’ quality of life; they become increasingly dependent on others	100.0%	Very high
	Ataluren (in addition to standard of care) delays the decline in nmDMD patients’ upper limb function, regardless of mobility status	95.0%	Very high
	Ataluren (in addition to standard of care) delays the decline of fine motor skills in nmDMD patients	95.0%	Very high

The threshold for consensus defined as > 66%, with consensus being defined as high at > 66% and very high at > 90% of respondents selecting agree or strongly agree

There is a large variability in upper limb function in adult patients with DMD [23]. Muscle strength and range of motion are factors strongly associated with upper limb function, and preserving these in patients with nmDMD would impact upper limb motor function outcome when they are adults [23].

Clinicians were asked to comment on six statements on upper limb function in the evaluation phase and the consensus was high across all of those statements. The consensus was 100% that the decline of upper limb function has a major impact on patients’ quality of life and that this can be delayed, regardless of mobility status, if they are treated with ataluren in addition to standard of care (Table 4).

### Pulmonary function

Death in nmDMD patients usually occurs as a result of cardiac or respiratory compromise [11]. Therefore, any impact that treatment with ataluren had on pulmonary function would be significant. A number (9) of statements about pulmonary function were included in the survey and there was a very high consensus (95–100%) across six of those statements and a high consensus across the other three (Table 5).

**Table 5:** There was a high or very high consensus regards the importance of delaying loss of pulmonary function and the positive role of ataluren

Theme	Statement	Overall Agreement	Consensus
Pulmonary function	Continuing the use of ataluren, in addition to the standard of care, in nmDMD patients when they lose ambulation delays the decline in pulmonary function	100.0%	Very high
	Maintaining patients’ pulmonary function means they experience fewer respiratory infections and may require less frequent hospitalisations	100.0%	Very high
	The ability of nmDMD patients to cough is maintained for longer with ataluren, in addition to standard of care	95.0%	Very high
	Ataluren, in addition to standard of care, delays the decline in nmDMD patients’ pulmonary function	95.0%	Very high
	Ataluren, in addition to standard of care, significantly delays the decline in nmDMD patients’ pulmonary function	80.0%	High
	Ataluren, in addition to standard of care, prolongs nmDMD patients’ ability to breathe independently	95.0%	Very high
	Ataluren, in addition to standard of care, significantly prolongs nmDMD patients’ ability to breathe independently	85.0%	High
	When nmDMD patients’ FVC falls below 60%, at latest, it becomes necessary to commence physiotherapy and/or screening for night-time ventilation	85.0%	High
	Patients that continue to receive ataluren, in addition to standard of care, after loss of ambulation are expected to have a delayed requirement for ventilation	95.0%	Very high

The threshold for consensus defined as > 66%, with consensus being defined as high at > 66% and very high at > 90% of respondents selecting agree or strongly agree

The results on pulmonary function indicated that there was a very high consensus that continuing the use of ataluren, in addition to the standard of care, in nmDMD patients when they lose ambulation delays the decline in pulmonary function.

### Cardiac function and duration of treatment with ataluren

As previously stated, death in nmDMD patients usually occurs as a result of cardiac or respiratory compromise and when patients are treated with assisted ventilation, cardiac function becomes the main determinant of survival [11]. Therefore, any impact that a treatment has on cardiac function would be significant. Clinicians evaluated four statements on cardiac function in the evaluation phase (Table 6).

**Table 6:** Clinicians evaluated statements on cardiac function and duration of treatment with ataluren and there was a high or very high consensus across the statements

Theme	Statement	Overall Agreement	Consensus
Cardiac function	It is logical to expect ataluren in nmDMD patients to have an effect on all muscles, including the cardiac muscle	95.0%	Very high
	It is logical to expect ataluren in addition to standard of care, to delay the onset of cardiac decline in patients with nmDMD	90.0%	High
	It is logical to expect ataluren, in addition to standard of care, to delay the onset of cardiomyopathy in patients with nmDMD	90.0%	High
	It is logical to expect ataluren, in addition to standard of care, to delay the decline in left ventricular ejection fraction with nmDMD	90.0%	High
Duration of treatment with ataluren	It is important to preserve the function of even small muscles in nmDMD patients	100.0%	Very high
	Treatment with ataluren should be continued as long as both the physician and nmDMD patients are both willing to continue treatment	90.0%	High
	As long as nmDMD patients still have functionally important muscles that can be influenced by ataluren they should continue to be treated	100.0%	Very high

The threshold for consensus defined as > 66%, with consensus being defined as high at > 66% and very high at > 90% of respondents selecting agree or strongly agree

Clinicians evaluated the four statements regards cardiac function and there was a high consensus across all. Indeed there was a very high consensus (95%) for the statement that ‘It is logical to expect ataluren in nmDMD patients to have an effect on all muscles, including the cardiac muscle’.

Clinicians evaluated three statements on the duration of treatment with ataluren and there was a very high consensus that for as long as nmDMD patients still have functionally important muscles that can be influenced by ataluren they should continue to be treated (Table 6).*Treatment with ataluren*.

Ataluren’s activity in patients with nmDMD has been demonstrated in clinical studies [10, 24–29] but the consensus amongst experienced clinicians in the evaluation phase agreed on its effectiveness in real-world clinical practice (Table 7).

**Table 7:** Clinicians were positive about the role of ataluren in the treatment of patients with nmDMD

Theme	Statement	Overall Agreement	Consensus
Ataluren	Ataluren is generally well tolerated	100.0%	Very high
	Ataluren (in addition to standard of care) delays disease progression in patients with nmDMD	100.0%	Very high
	Patients receiving treatment with ataluren appear to have more energy	95.0%	Very high
	Patients receiving treatment with ataluren seem to better manage daily situations	90.0%	High
	Patients receiving treatment with ataluren appear to have a better overall quality of life	95.0%	Very high
	Ataluren (in addition to standard of care) significantly delays the decline in muscle function in patients with nmDMD	75.0%	High

The threshold for consensus defined as > 66%, with consensus being defined as high at > 66% and very high at > 90% of respondents selecting agree or strongly agree

## Discussion

The importance of the loss of motor function to patients with nmDMD is widely acknowledged and the progressive loss of each function is usually irreversible [19]. As detailed in the results of this modified Delphi methodology, a number of statements relating to the progression of nmDMD were included in the evaluation survey and there was very high consensus across many of them. There was consensus that the speed of progression of nmDMD is variable and individual to each patient, and that the earlier nmDMD patients are diagnosed and treatment initiated, the greater the delay in muscle decline. There was also agreement that proximal lower limb muscles are amongst the first to decline in nmDMD leading to loss of ambulation.

The clinicians who participated in this study agreed (100%) that there is life beyond loss of ambulation. There are still lots of important functions of the muscles such as being able to use the hands and arms, fine motor skills and respiratory muscles – all these functions should be maintained for as long as possible. There was also agreement that delaying the loss of ambulation may reduce the development of scoliosis, the decline in respiratory function and the decline of upper limb function. These all have a significant impact on nmDMD patients’ quality of life.

It is acknowledged that death in nmDMD patients usually occurs as a result of cardiac or respiratory compromise [11]. Therefore, any impact that a treatment has on pulmonary and/or cardiac function would be significant. There was very high consensus (95%) from the clinicians continuing the use of ataluren, in addition to standard of care, in nmDMD patients when they lose ambulation delays the decline in pulmonary function, thereby maintaining the ability of those patients to cough and breathe independently, delaying the requirement for ventilation. Respondents also agreed (95%) with the statement that ’It is logical to expect ataluren, in addition to the standard of care, to have an effect on all muscles, including the cardiac muscles’.

To delay the loss of function in nmDMD patients, treatments that do more than just address symptoms (the standard of care) are required. Ataluren is a disease-modifying treatment [9, 10] and clinicians who participated in the evaluation phase agreed that treatment with ataluren, in addition to the standard of care, has a positve impact on delaying loss of ambulation, the loss of fine motor skills, the decline in pulmonary function and the development of scoliosis. There was also agreement (100%) that treatment with ataluren should be continued as long as both the physician and nmDMD patients are both willing to continue treatment.

Findings of evaluation phase suggest that treatment with ataluren should be initiated as soon as possible to delay disease progression and allow patients to remain ambulatory for as long as possible.

It should be noted that Delphi panels are based on the respondent’s clinical experience and opinion and therefore the opinion of the respondents in the Delphi study and the published data may not necessarily align, as is the case with the statement “Non-ambulatory nmDMD patients are less likely to develop scoliosis if they continue treatment with ataluren (in addition to standard of care) after loss of ambulation”, where 75% of respondents agreed with this statement, but the published literature does not reflect this opinion.

A possible limitation of this study is the use of the 4-point Likert scale as this forces either agreement or disagreement with each statement (strongly disagree, disagree, agree, or strongly agree to indicate level of agreement) and could potentially lead to some level of positive bias as there is not an option to ”neither agree nor disagree”. A second limitation of this study is that as we did not have GDPR permission to share details of the respondents from the exploration phase with an external third party, it was therefore not possible for clinicians that were interviewed for the exploration phase to be removed from the recruitment list that TREAT-NMD generated for the evaluation phase. A subsequent retrospective review of respondents revealed that 4 clinicians responded to both the exploration and evaluation phases.

## Conclusions

There is an unmet need for a treatment that can be used in patients with nmDMD who have already progressed to the non-ambulatory phase that delays disease progression, maintains some essential functions and has a good tolerability profile.

Clinical experience and expert opinion suggest that ataluren use in both ambulatory and non-ambulatory DMD patients has a positive impact on patient outcomes and quality of life, and should be part of standard of care treatment in ambulatory and non-ambulatory patients.

This modified Delphi methodology work found that treatment with ataluren is believed by experts to be beneficial in both ambulatory and non-ambulatory patients and, as long as the treatment is effective, should be continued for as long as both the physician and nmDMD patient are both willing to continue treatment.

This consensus could drive clinicians to review their current practice and make appropriate clinical decisions in the best interests of their individual DMD patients. However, what is practical to implement is often restricted by European and national regulations and guidelines.

### Electronic supplementary material

Below is the link to the electronic supplementary material.

Supplementary Material 1

Supplementary Material 2

Supplementary Material 3

Supplementary Material 4

## Data Availability

The raw data for the evaluation phase can be accesses in Appendix D.

## Abbreviations

DMD: Duchenne muscular dystrophy
FVC: Forced vital capacity
nmDMD: nonsense mutation Duchenne muscular dystrophy
SmPC: Summary of Product Characteristics
