# Supplementary material for: The role of ataluren in the treatment of ambulatory and non-ambulatory children with nonsense mutation duchenne muscular dystrophy - a consensus derived using a modified Delphi methodology in Eastern Europe, Greece, Israel and Sweden
Source: BMC Neurol. 2024 Feb 21;24:73. doi: 10.1186/s12883-024-03570-x (PMC10880248; doi:10.1186/s12883-024-03570-x)
Supplement: Supplementary file 1 — Supplementary Material 1 [file 12883_2024_3570_MOESM1_ESM.pdf]

Appendix A

DMD Virtual Delphi – Exploration phase

Sections and expected duration

Interviewer note: the table below is for information only, not described as part of interview introduction.

Section	Expected duration (minutes)
Introduction	5
Background information	10
Clinical experience with ataluren	15
Clinical interpretation of ataluren data in non- ambulatory patients and opinions on the clinical value	35
Value & reimbursement of ataluren in non-ambulatory patients	20
Closing comments (interviewer)	5
**Total**	**90**

Introduction

Interviewer notes: introduce each other, confirm consultation structure and project compliance, housekeeping

Expected duration: 5 minutes

Objective: The objective of the project is to gain an understanding of physicians experience with ataluren in non-ambulatory Duchenne Muscular Dystrophy (DMD) patients, your clinical interpretation of available evidence and the value of ataluren in non-ambulatory patients.

Duration: The expected duration of this consultation is approximately 90 minutes.

Compliance: We comply with all relevant codes of conduct including Association of the British Pharmaceutical Industry (ABPI), Data Protection Act, Market Research Society, British Healthcare Business Intelligence Association (BHBIA).

Sponsor: This consultation is being conducted by The MASS Team on behalf of PTC Therapeutics International Limited.

Anonymised research: As part of this project, your comments will be reported back to PTC Therapeutics International Limited in a consolidated, anonymised fashion. The identity of respondents maybe revealed, but not their individual responses. Of course, during participation, you have the right to refuse to answer any questions or withdraw altogether.

Right to withdraw: You have the right to withdraw from this consultation at any time or to decline to answer any particular questions you do not feel comfortable answering or if you feel the answer to the question would disclose confidential information.

Fee: Your fee for participation has been outlined in the contract.

GDPR: It is now a requirement of data protection law (specifically the GDPR) that the data controller is revealed to you. The data controller is the pharmaceutical company, PTC Therapeutics International Limited. You can find the PTC Therapeutics Privacy Notice on their website.

Adverse events: We are required to pass on to PTC Therapeutics International Limited details of

adverse events/product complaints pertaining to their products that are mentioned during the course of this consultation. Although what you say will, of course, be treated in confidence, should you raise during the discussion an adverse event or product complaint in a specific patient, or group of patients, we will need to report this even if it already has been reported by you directly to PTC Therapeutics or the regulatory authorities. In such a situation, you will be asked whether or not you are willing to waive confidentiality specifically in relation to that adverse event / product complaint. Everything else you say during the course of the interview will continue to remain anonymous if you so wish. Are you happy to participate with the consultation on this basis?

Audio recording: Any audio recording is for the purpose of aiding the interviewer's own notes only, it will not be shared with anyone else within The MASS Team or any external third party, including PTC Therapeutics International Limited. Any audio recording will be destroyed upon the completion of this project. Are you happy for me to start recording our conversation on this basis?

Interviewers note: Start recording (assuming agreement)

There are no right or wrong answers, we are purely interested in your honest views and opinions.

Background information – Expected duration 10 minutes

Interviewer note: The purpose of this section is to understand more about the role of the respondent

1. Could you briefly describe your responsibilities within your role?

2. How often do you see patients with Duchenne Muscular Dystrophy (DMD)?

Interviewer note: for the purposes of this interview Duchenne Muscular Dystrophy will be referred to as DMD from this point on.

3. How many Duchenne Muscular Dystrophy (DMD) patients are managed at your centre?

Interviewer prompt: What percentage of the total DMD patients at your centre have DMD due to a nonsense mutation?

4. How many of your nmDMD (nonsense mutation DMD) patients are currently ambulatory?

Interviewer prompt: What percentage of these are currently receiving therapy with ataluren?

5. How many of your nmDMD patients are currently non ambulatory?

Interviewer prompts: How many of your patients have transition to being non- ambulatory in the last 12 months? What percentage of these are currently receiving therapy with ataluren?

6. What tools/measurements do you use to determine if a patient is defined as ambulatory or non-ambulatory?

Interviewer prompts: What are the key measurements and values that inform your decision on the patient's ambulatory status?

Clinical experience with ataluren – Expected duration 15 minutes

Interviewers note: The purpose of this section is to understand the physicians clinical experience of ataluren and their perceptions of the benefits of ataluren in non-ambulatory patients

7. Can you please describe the clinical benefits that your ambulatory patients have gained from treatment with ataluren?

Interviewer prompts: Do you think ataluren has delayed the decline in your patient's ability to ascend stairs? Do you think ataluren has delayed the decline in your patient's ability to descend stairs?

8. As DMD progresses, patients will eventually become non-ambulatory, can you please describe the clinical benefits that your non-ambulatory patients have gained from continued treatment with ataluren?

Interviewer prompts: How have these observations compared with your expected disease progression for these patients?

9. How has continued treatment with ataluren has delayed the decline in your non- ambulatory patient's upper limb function?

Interviewer prompts: How do you measure this clinically, Performance of Upper Limb (PUL)? MyoGrip test? MyoPinch test? MoviPlate test? MyoWrist test? Muscle Function Measure (MFM)? If so, what outcomes have you observed? How have these observations compared with your expected disease progression for these patients?

10. How has continued treatment with ataluren has delayed the decline in your non- ambulatory patient's pulmonary function?

Interviewer prompts: How do you measure this clinically, Forced Vital capacity (FVC)? Forced Expiratory Fraction (FEV)? Peak Expiratory Flow (PEF)? Peak Cough Flow (PCF)? If so, what outcomes have you observed? How have these observations compared with your expected disease progression for these patients?

11. How has continued treatment with ataluren has delayed the decline in your non- ambulatory patient's cardiac function?

Interviewer prompts: How do you measure this clinically, Left Ventricular Ejection Fraction (LVEF)? Shortening Fraction (SF)? If so, what outcomes have you observed? How have these observations compared with your expected disease progression for these patients?

12. Has treatment with ataluren impacted your non-ambulatory patient's quality of life?

Interviewer prompts: How do believe you believe the quality of life has been impacted? Do you measure this clinically, if so, what outcomes have you observed? How have these observations compared with your expected disease progression for these patients?

13. Are there any other points you would like to make about the clinical benefits of ataluren you have observed in your non-ambulatory patients before we move on from this section of the discussion?

Clinical interpretation of ataluren data in non-ambulatory patients and opinions on the clinical value – Expected duration 35 minutes

Interviewers note: In this section we will be showing respondents clinical data from non-ambulatory patients treated with ataluren. The purpose of the section is to determine the respondent's clinical interpretation of the data and the value that they believe this will deliver to their patients. Stimulus materials will be shown to the respondent using the screen share facility in Microsoft Teams and are not to be sent (either physically or digitally) to the respondent. The stimulus material is identified by the number in the bottom right-hand corner of the slide.

Interviewers note: Please screen share with the respondent and present slide 1, confirm they can see slide 1 and read the disclaimer on slide 1.

In this section of our discussion, I am going to show you some data on ataluren, give you a few moments to review this data and then ask for your views and opinions on the data.

Interviewers note: Present **slide 2 (Ataluren mechanism of action)** from the stimulus material, confirm that they can see the data and give the respondent a few minutes to read and review the slide.

14. What is your initial reaction to this data?

Interviewers prompts: What is your clinical interpretation of this data? How does this data relate to your non-ambulatory patients?

15. What does this data tell you about the value of ataluren?

Interviewers prompts: What does this data tell you about the value of ataluren in non-ambulatory patients? In your opinion, is the value in non-ambulatory patients different to ambulatory or the same?

16. Is there anything else you would like to add about this data and the value to your non-ambulatory patients?

Interviewers note: Move stimulus material on to **slide 3 (Delaying the loss of ambulation delays respiratory complications)** , confirm that they can see the data and give the respondent a few minutes to read and review the slide.

17. What is your initial reaction to this data?

Interviewers prompts: What is your clinical interpretation of this data? How does this data relate to your non-ambulatory patients?

18. What does this data tell you about the value of ataluren?

Interviewers prompts: What does this data tell you about the value of ataluren in non-ambulatory patients? In your opinion, is the value in non-ambulatory patients different to ambulatory or the same?

19. Is there anything else you would like to add about this data and the value to your non-ambulatory patients?

Interviewers note: Move stimulus material on to **slide 4 (Age at predicted FVC <60%)**, confirm that they can see the data and give the respondent a few minutes to read and review the slide.

20. What is your initial reaction to this data?

Interviewers prompts: What is your clinical interpretation of this data? How does this data relate to your non-ambulatory patients?

21. What does this data tell you about the value of ataluren?

Interviewers prompts: What does this data tell you about the value of ataluren in non-ambulatory patients? In your opinion, is the value in non-ambulatory patients different to ambulatory or the same? Having seen the effect of ataluren on pulmonary outcomes in non-ambulatory patients in the STRIDE registry compared to matched patients from the Cooperative International Neuromuscular Research Group Duchenne Natural History Study, would you expect to see any corresponding clinical effect on patient's Upper limb function?

22. Is there anything else you would like to add about this data and the value to your non-ambulatory patients?

Interviewers note: Move stimulus material on to **slide 5 (Age at predicted FVC >30%)**, confirm that they can see the data and give the respondent a few minutes to read and review the slide.

23. What is your initial reaction to this data?

Interviewers prompts: What is your clinical interpretation of this data? How does this data relate to your non-ambulatory patients?

24. What does this data tell you about the value of ataluren?

Interviewers prompts: What does this data tell you about the value of ataluren in non-ambulatory patients? In your opinion, is the value in non-ambulatory patients different to ambulatory or the same? Having seen the effect of ataluren on pulmonary outcomes in non-ambulatory patients in the STRIDE registry compared with matched patients from the Cooperative International Neuromuscular Research Group Duchenne Natural History Study, would you expect to see any corresponding clinical effect on patient's Upper limb function?

25. Is there anything else you would like to add about this data and the value to your non-ambulatory patients?

Benefits of ataluren in non-ambulatory patients – Expected duration 20 minutes Interviewers note: The purpose of this section is to understand if the reimbursement of ataluren is different in non-ambulatory patients and ambulatory patients in the respondent's country and, if different, their views on this.

26. What do you see as the benefits from continuing use of ataluren in patients that have progressed to the non-ambulatory phase?

Interviewers prompts: What specific outcomes would you be expecting to achieve through continued use of ataluren?

27. Based on your clinical experience, what benefits would you expect to clinically observe in your patient's upper limb function from continuing use of ataluren in patients that have progressed to the non-ambulatory phase?

Interviewers prompts: What specific upper limb outcomes would you be expecting to achieve through continued use of ataluren?

28. Based on your clinical experience, what benefits would you expect to clinically observe in your patient's pulmonary function from continuing use of ataluren in patients that have progressed to the non-ambulatory phase?

Interviewers prompts: What specific pulmonary outcomes would you be expecting to achieve through continued use of ataluren?

29. Based on your clinical experience, what benefits would you expect to clinically observe in your patient's cardiac function from continuing use of ataluren in patients that have progressed to the non-ambulatory phase?

Interviewers prompts: What specific cardiac outcomes would you be expecting to achieve through continued use of ataluren?

30. Ideally, how long would you want to continue use of ataluren in your non-ambulatory patients?

Interviewers prompts: Would you ever see a time when you would want to stop treatment with ataluren in non-ambulatory patients? (if yes) When would you stop treatment with ataluren? What milestones or clinical measures would inform your decision to stop treatment with ataluren?

31. How would you summarise the overall value of ataluren in non-ambulatory patients?

Interviewers prompts: If you were summarising the value of ataluren to your local payer, would you keep this summary the same or change it at all?

32. Is there anything else you would like to add that we have not covered today? (Time permitting)

Closing comments (interviewer) – Expected duration of section: 5

Interviewer note: Purpose of section to provide payment procedure and next steps.

- The MASS Team are currently conducting a number of consultations with experts in DMD, such as yourself, with the intensions of collating common themes and opinions. These themes may then be taken forward to a Delphi process (which is a recognised process for achieving consensus) with the objective of establishing which themes the wider DMD community are in consensus with.

- Would you be interested in being involved with such a process?

- Would you be happy for the MASS Team to contact you in the future in connection with a Delphi process?

- Procedure for payment of honoraria (interviewer to send honorarium payment form, respondent to populate bank details and return for processing)

- Thank for respondent for their time
