# Supplementary material for: The role of ataluren in the treatment of ambulatory and non-ambulatory children with nonsense mutation duchenne muscular dystrophy - a consensus derived using a modified Delphi methodology in Eastern Europe, Greece, Israel and Sweden
Source: BMC Neurol. 2024 Feb 21;24:73. doi: 10.1186/s12883-024-03570-x (PMC10880248; doi:10.1186/s12883-024-03570-x)
Supplement: Supplementary file 2 — Supplementary Material 2 [file 12883_2024_3570_MOESM2_ESM.pdf]

Appendix B

Evaluation Questionnaire

Thank you for taking part in this important study to gain consensus from the Duchenne Muscular Dystrophy clinical community.

Using the Delphi methodology, this panel aim to gain consensus on:

- **Physicians' own experience of use of ataluren in Nonsense Mutation Duchenne Muscular Dystrophy (nmDMD) patients**
- **Physicians' opinions on the value of ataluren in ambulatory nmDMD patients**
- **Physicians' opinions on the value of ataluren in non-ambulatory nmDMD patients**

This questionnaire contains a number of statements that have been produced following in-depth interviews with twelve consultant paediatric neurologists across nine countries. The statements have then been reviewed and approved by an expert panel of consultant paediatric neurologists.

This questionnaire will present you with a range of statements and ask you to rate them on a four-point scale, from strongly disagree to strongly agree. There is also an area for comments should you want to briefly explain your rating. The questionnaire should take no more than 30 minutes to complete. Once you have completed the questionnaire you will be sent an honorarium claim form, via email, which will enable you to claim a modest honorarium for the time taken to complete this questionnaire.

The aspirational objective of this study is to publish the results in a peer reviewed journal.

If you have any questions, please contact Craig Dixon at craig@mass-team.com

Thank you for your participation in this important study

Dr Thomas Sejersen
Dr Tanja Golli
Dr Lenka Jurikova

Please note: This study is being administered by the MASS Team. This project has been funded by a pharmaceutical company, but the contents of this questionnaire have been produced independently by Dr Tanja Golli, Dr Lenka Jurikova, Dr Thomas Sejersen and the MASS

To ensure the Delphi Panel only collects responses from physicians with the appropriate experience can you please answer the two questions below before proceeding. Thank you

* 1. Do you treat, or have you ever treated, patients with Duchenne Muscular Dystrophy due to a nonsense mutation (nmDMD)?

- ◯ Yes
- ◯ No

* 2. Do you have clinical experience with ataluren in nmDMD patients?

- ◯ Yes
- ◯ No

Respondents details

* 3. **Please enter your full name:**

4. **Which country are you based in?**

* 5. **Address of the centre you work at:**

* 6. **What is your position at this centre?**

Please note: If your position is not listed please do not proceed with this questionaire

- ◯ Consultant Neurologist
- ◯ Neurologist
- ◯ Consultant Paediatric Neurologist
- ◯ Paediatric Neurologist
- ◯ Other (please specify)

* 7. **Please enter your email address:**

This will be used to contact you for any subsequent rounds of the Delphi process. The MASS Team will share your name and email address with the TREAT-NMD Global Registries Manger and the registry you heard about this survey from, so they can ensure you are removed from any future emails sent to remind people about this questionnaire.

By entering your email address, you are giving permission for the MASS Team to contact you and store your contact details for the purposes of this study only. This data will be stored in EU with the MASS Team, TREAT-NMD and your local registry classified as data processors, the data controller is the project sponsor, PTC Therapeutics International. The MASS Team will irretrievably delete your data once this project is completed. This research will comply with UK Data Protection Act 2018/GDPR. You have the right to access your data, the right to erasure of your data and the right to rectification of any of your personal information you think in inaccurate. Please contact us at info@mass-team.com if you wish to make a request.

Progression of nmDMD

Please rate the following statements using the following options:

- **Strongly disagree**
- **Disagree**
- **Agree**
- **Strongly agree**

Each statement also has a comments box should you wish to briefly explain your rating - Populating this box is especially important for us to understand your response if you disagree or strongly disagree with the statement

* 8. The speed of progression of nmDMD is variable and individual to the patient

Strongly disagree	Disagree	Agree	Strongly agree
○	○	○	○

Comments

* 9. The earlier nmDMD patients are diagnosed and treatment initiated, the greater the delay in muscle decline

Strongly disagree	Disagree	Agree	Strongly agree
○	○	○	○

Comments

* 10. Proximal lower limb muscles are are the amongst the first to decline in nmDMD leading to loss of ambulation

Strongly disagree	Disagree	Agree	Strongly agree
○	○	○	○

Comments

* 11. Decline in cardiac and pulmonary function are two of the major causes of death in nmDMD

Strongly disagree	Disagree	Agree	Strongly agree
○	○	○	○

Comments

Ataluren

Please rate the following statements using the following options:

Strongly disagree
Disagree
Agree
Strongly agree

Each statement also has a comments box should you wish to briefly explain your rating - Populating this box is especially important for us to understand your response if you disagree or strongly disagree with the statement

* 12. Ataluren is generally well tolerated

Strongly disagree	Disagree	Agree	Strongly agree
○	○	○	○

Comments

* 13. Ataluren (in addition to standard of care) delays disease progression in patients with nmDMD

Strongly disagree	Disagree	Agree	Strongly agree
○	○	○	○

Comments

* 14. Patients receiving treatment with ataluren appear to have more energy

Strongly disagree	Disagree	Agree	Strongly agree
○	○	○	○

Comments

* 15. Patients receiving treatment with ataluren seem to better manage daily situations

Strongly disagree	Disagree	Agree	Strongly agree
○	○	○	○

Comments

* 16. Patients receiving treatment with ataluren appear to have a better overall quality of life

Strongly disagree	Disagree	Agree	Strongly agree
○	○	○	○

Comments

* 17. Ataluren (in addition to standard of care) significantly delays the decline in muscle function in patients with nmDMD

Strongly disagree	Disagree	Agree	Strongly agree
○	○	○	○

Comments

Loss of ambulation

Please rate the following statements using the following options:

Strongly disagree
Disagree
Agree
Strongly agree

Each statement also has a comments box should you wish to briefly explain your rating - Populating this box is especially important for us to understand your response if you disagree or strongly disagree with the statement

* 18. Ataluren (in addition to standard of care) significantly delays the loss of ambulation in patients with nmDMD

Strongly disagree	Disagree	Agree	Strongly agree
○	○	○	○

Comments

* 19. Ataluren (in addition to standard of care) is expected to result in the same treatment effect in each surviving muscle fibre irrespective of the nmDMD patients' ambulatory status

Strongly disagree	Disagree	Agree	Strongly agree
○	○	○	○

Comments

* 20. If a nmDMD patient receiving ataluren loses ambulation, they should continue treatment with ataluren

Strongly disagree	Disagree	Agree	Strongly agree
○	○	○	○

Comments

* 21. There is life beyond loss of ambulation. There are still lots of important functions of the muscles, such as being able to use the hands and arms, fine motor skills and respiratory muscles – all these functions should be maintained for as long as possible

Strongly disagree	Disagree	Agree	Strongly Agree
○	○	○	○

Comments

* 22. Delaying the loss of ambulation in patients with nmDMD may reduce the development of scoliosis

Strongly disagree	Disagree	Agree	Strongly agree
○	○	○	○

Comments

* 23. Delaying the loss of ambulation in patients with nmDMD delays the decline of respiratory function

Strongly disagree	Disagree	Agree	Strongly agree
◯	◯	◯	◯

Comments

* 24. Delaying the loss of ambulation in patients with nmDMD is related to the decline of upper limb function

Strongly disagree	Disagree	Agree	Strongly agree
◯	◯	◯	◯

Comments

Scoliosis

Please rate the following statements using the following options:

Strongly disagree
Disagree
Agree
Strongly agree

Each statement also has a comments box should you wish to briefly explain your rating - Populating this box is especially important for us to understand your response if you disagree or strongly disagree with the statement

* 25. Development of scoliosis has a detrimental impact on patients' pulmonary function

Strongly disagree	Disagree	Agree	Srongly agree
◯	◯	◯	◯

Comments

* 26. nmDMD patients treated with ataluren (in addition to standard of care) are less likely to develop scoliosis

Strongly disagree	Disagree	Agree	Strongly Agree
○	○	○	○

Comments

* 27. Non-ambulatory nmDMD patients are less likely to develop scoliosis if they continue treatment with ataluren (in addition to standard of care) after loss of ambulation

Strongly disagree	Disagree	Agree	Strongly Agree
○	○	○	○

Comments

Upper limb function

Please rate the following statements using the following options:

Strongly disagree
Disagree
Agree
Strongly agree

Each statement also has a comments box should you wish to briefly explain your rating - Populating this box is especially important for us to understand your response if you disagree or strongly disagree with the statement

* 28. Delaying the decline of muscle function in patients' upper limbs helps to maintain independence

Strongly disagree	Disagree	Agree	Strongly agree
○	○	○	○

Comments

* 29. Delaying in the decline in fine motor skills also enables patients in wheelchairs to continue to be as independent as possible

Strongly disagree	Disagree	Agree	Strongly agree
◯	◯	◯	◯

Comments

* 30. Delaying the decline of upper limb strength enables non-ambulatory patients to transfer from their wheelchair to the toilet, maintain intimate hygiene, retain independence and protect their quality of life

Strongly disagree	Disagree	Agree	Strongly agree
◯	◯	◯	◯

Comments

* 31. Decline of upper limb function has a major impact on patients' quality of life; they become increasingly dependant on others

Strongly disagree	Disagree	Agree	Strongly agree
◯	◯	◯	◯

Comments

* 32. Ataluren (in addition to standard of care) delays the decline in nmDMD patients' upper limb function, regardless of mobility status

Strongly disagree	Disagree	Agree	Strongly agree
◯	◯	◯	◯

Comments

* 33. Ataluren (in addition to standard of care) delays the decline of fine motor skills in nmDMD patients

Strongly disagree	Disagree	Agree	Strongly agree
○	○	○	○

Comments

Pulmonary function

Please rate the following statements using the following options:

Strongly disagree
Disagree
Agree
Strongly agree

Each statement also has a comments box should you wish to briefly explain your rating - Populating this box is especially important for us to understand your response if you disagree or strongly disagree with the statement

* 34. Continuing the use of ataluren, in addition to standard of care, in nmDMD patients when they lose ambulation delays the decline in pulmonary function

Strongly disagree	Disagree	Agree	Strongly agree
○	○	○	○

Comments

* 35. Maintaining patients' pulmonary function means they experience fewer respiratory infections and may require less frequent hospitalisations

Strongly disagree	Disagree	Agree	Strongly agree
○	○	○	○

Comments

* 36. The ability of nmDMD patients to cough is maintained for longer with ataluren, in addition to standard of care

Strongly disagree	Disagree	Agree	Strongly agree
○	○	○	○

Comments

* 37. Ataluren, in addition to standard of care, delays the decline in nmDMD patients' pulmonary function

Strongly disagree	Disagree	Agree	Strongly agree
○	○	○	○

Comments

* 38. Ataluren, in addition to standard of care, significantly delays the decline in nmDMD patients' pulmonary function

Strongly disagree	Disagree	Agree	Strongly agree
○	○	○	○

Comments

* 41. When nmDMD patients' FVC falls below 60%, at latest, it becomes necessary to commence physiotherapy and/or screening for night-time ventilation

Strongly disagree	Disagree	Agree	Strongly agree
◯	◯	◯	◯

Comments

* 42. Patients that continue to receive ataluren, in addition to standard of care, after loss of ambulation are expected to have a delayed requirement for ventilation

Strongly disagree	Disagree	Agree	Strongly agree
◯	◯	◯	◯

Comments

Cardiac function

Please rate the following statements using the following options:

Strongly disagree
Disagree
Agree
Strongly agree

Each statement also has a comments box should you wish to briefly explain your rating - Populating this box is especially important for us to understand your response if you disagree or strongly disagree with the statement

* 43. It is logical to expect ataluren in nmDMD patients to have an effect on all muscles, including the cardiac muscle

Strongly disagree	Disagree	Agree	Strongly agree
◯	◯	◯	◯

Comments

* 44. It is logical to expect Ataluren, in addition to standard of care, to delay the onset of cardiac decline in patients with nmDMD

Strongly disagree	Disagree	Agree	Strongly agree
◯	◯	◯	◯

Comments

* 45. It is logical to expect Ataluren, in addition to standard of care, to delay the onset of cardiomyopathy in patients with nmDMD

Strongly disagree	Disagree	Agree	Strongly agree
◯	◯	◯	◯

Comments

* 46. It is logical to expect Ataluren, in addition to standard of care, to delay the decline in left ventricular ejection fraction in patients with nmDMD

Strongly disagree	Disagree	Agree	Strongly agree
◯	◯	◯	◯

Comments

Duration of treatment with ataluren

Please rate the following statements using the following options:

Strongly disagree
Disagree
Agree
Strongly agree

Each statement also has a comments box should you wish to briefly explain your rating - Populating this box especially important for us to understand your response if you disagree or strongly disagree with the statement

* 47. It is important to preserve the function of even small muscles in nmDMD patients

Strongly disagree	Disagree	Agree	Strongly agree
◯	◯	◯	◯

Comments

* 48. Treatment with ataluren should be continued as long as both the physician and nmDMD patient are both willing to continue treatment

Strongly disagree	Disagree	Agree	Strongly Agree
◯	◯	◯	◯

Comments

* 49. As long as nmDMD patients still have functionally important muscles that can be influenced by ataluren they should continue to be treated

Strongly disagree	Disagree	Agree	Strongly agree
◯	◯	◯	◯

Comments

Thank you

Thank you for taking the time to complete this Delphi Questionaire.

50. Please enter your details below to claim an honorarium of £75 for your time completing this questionnaire. Honorarium claim forms will be emailed for you to complete and return when the questionnaire closes.

Name ____________________

Email Address ____________________
