# Supplementary material for: The role of ataluren in the treatment of ambulatory and non-ambulatory children with nonsense mutation duchenne muscular dystrophy - a consensus derived using a modified Delphi methodology in Eastern Europe, Greece, Israel and Sweden
Source: BMC Neurol. 2024 Feb 21;24:73. doi: 10.1186/s12883-024-03570-x (PMC10880248; doi:10.1186/s12883-024-03570-x)
Supplement: Supplementary file 3 — Supplementary Material 3 [file 12883_2024_3570_MOESM3_ESM.pdf]

Evaluation phase full results dataset

Consensus Statements
# – Progression of nmDMD

The speed of progression of nmDMD is variable and individual to the patient

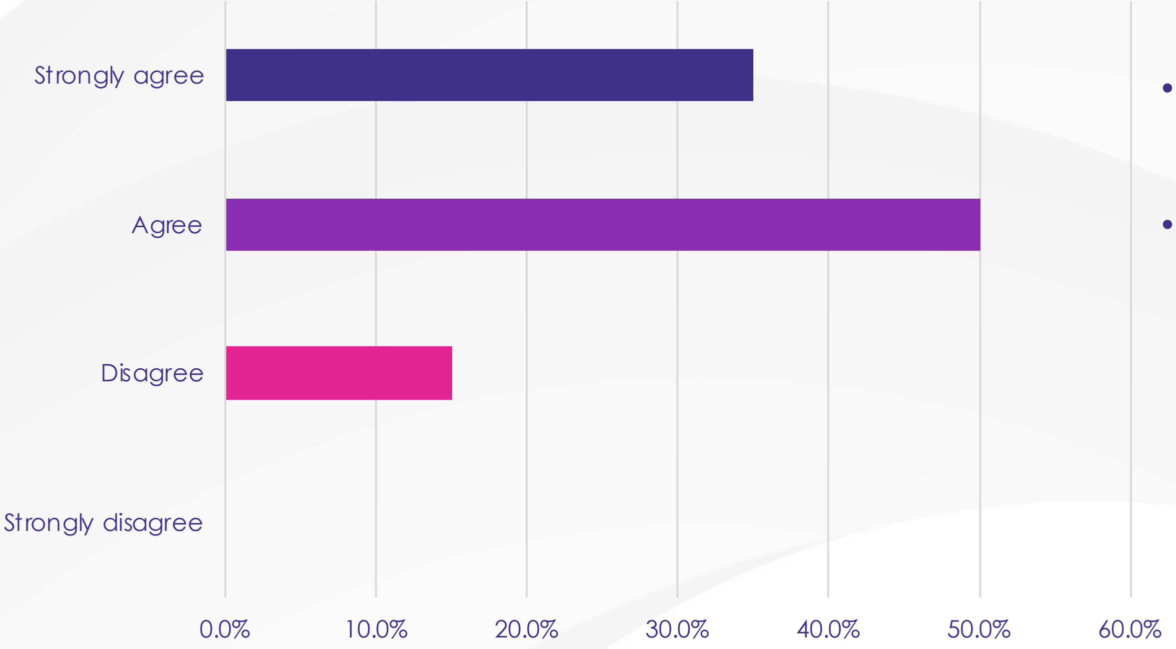

Comments

- in our patient similar age of losing of ambulation in patient nm with DMD
- variability is limited

Overall agreement 85%

The earlier nmDMD patients are diagnosed and treatment initiated, the greater the delay in muscle decline

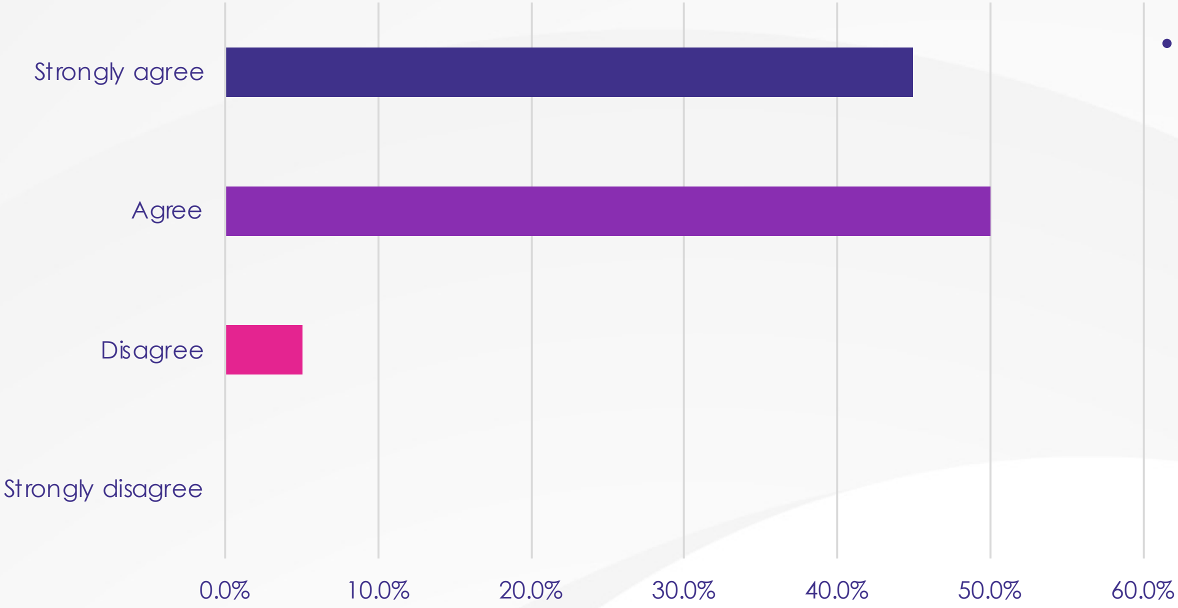

Comments

- it seems to be, we need more time to evaluate this

Overall agreement 95%

Proximal lower limb muscles are the amongst the first to decline in nmDMD leading to loss of ambulation

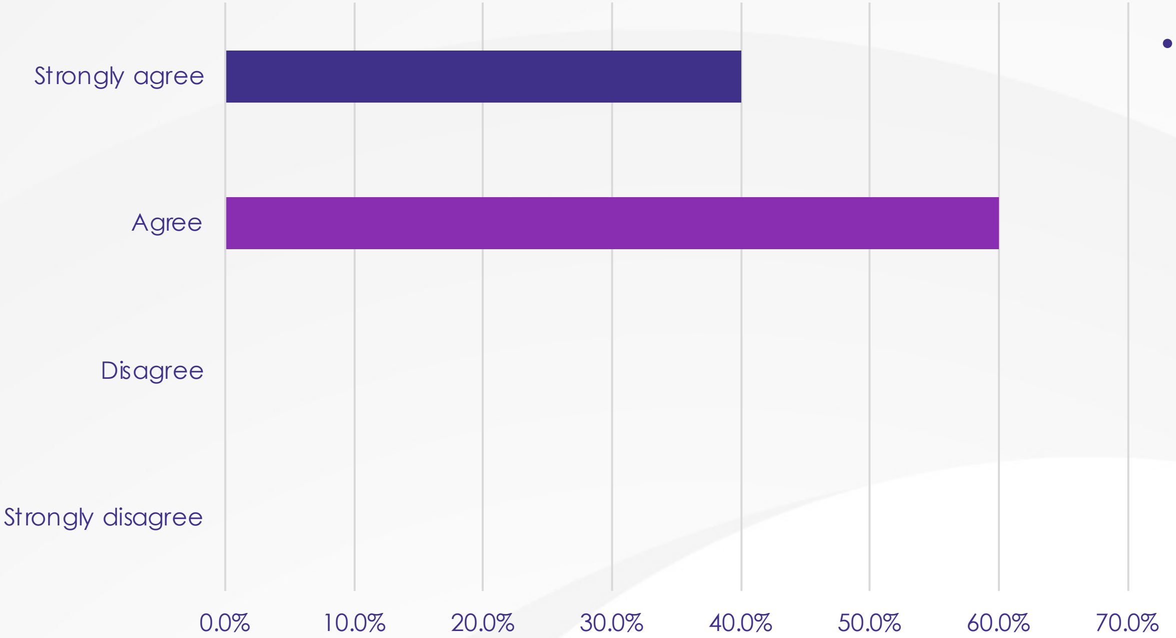

Comments

- This we see in all our DMD patient not just in patient with nmDMD

Overall agreement 100%

Decline in cardiac and pulmonary function are two of the major causes of death in nmDMD

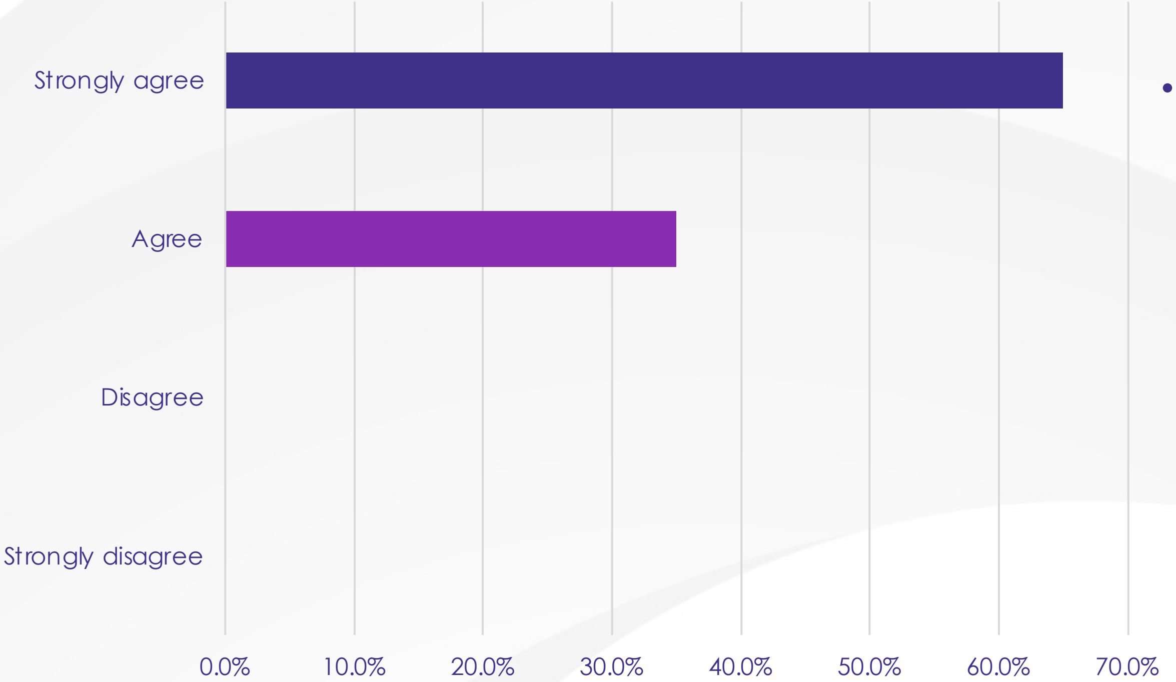

Comments

- Yes in our patient mostly cardiac arrest or respiratory failure is cause of death

Overall agreement 100%

Consensus Statements – Ataluren

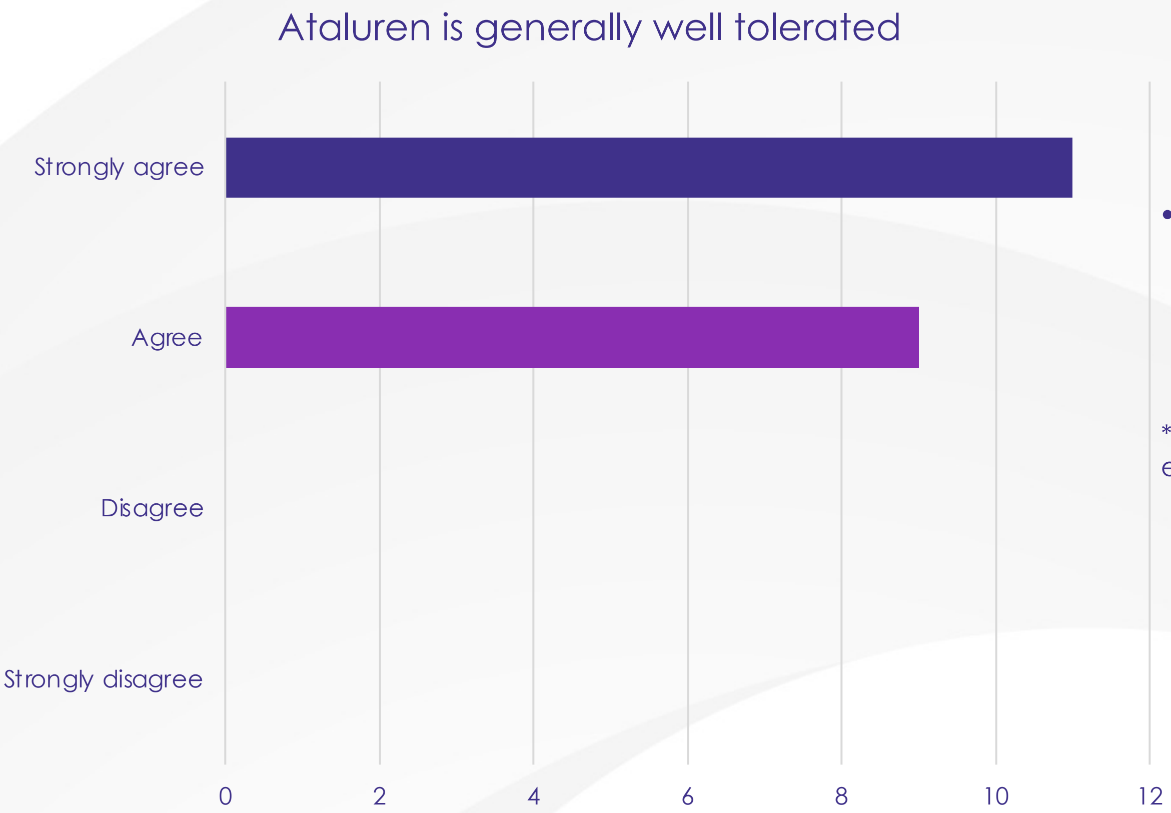

Comments

- We have just one patient with side effect vomiting*

Overall agreement 100%

*This comment was reported as an adverse event to PTC therapeutics international

Ataluren (in addition to standard of care) delays disease progression in patients with nmDMD

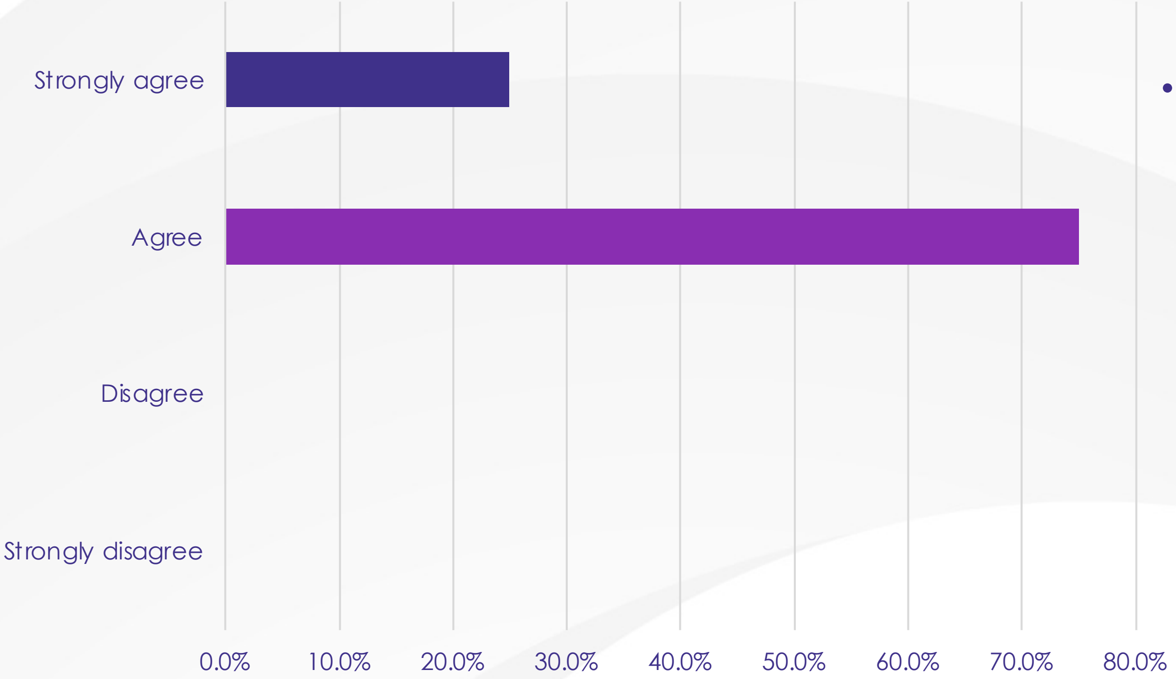

Comments

- It seems to be true our oldest ambulatory patient is 18 years old

Overall agreement 100%

Patients receiving treatment with ataluren appear to have more energy

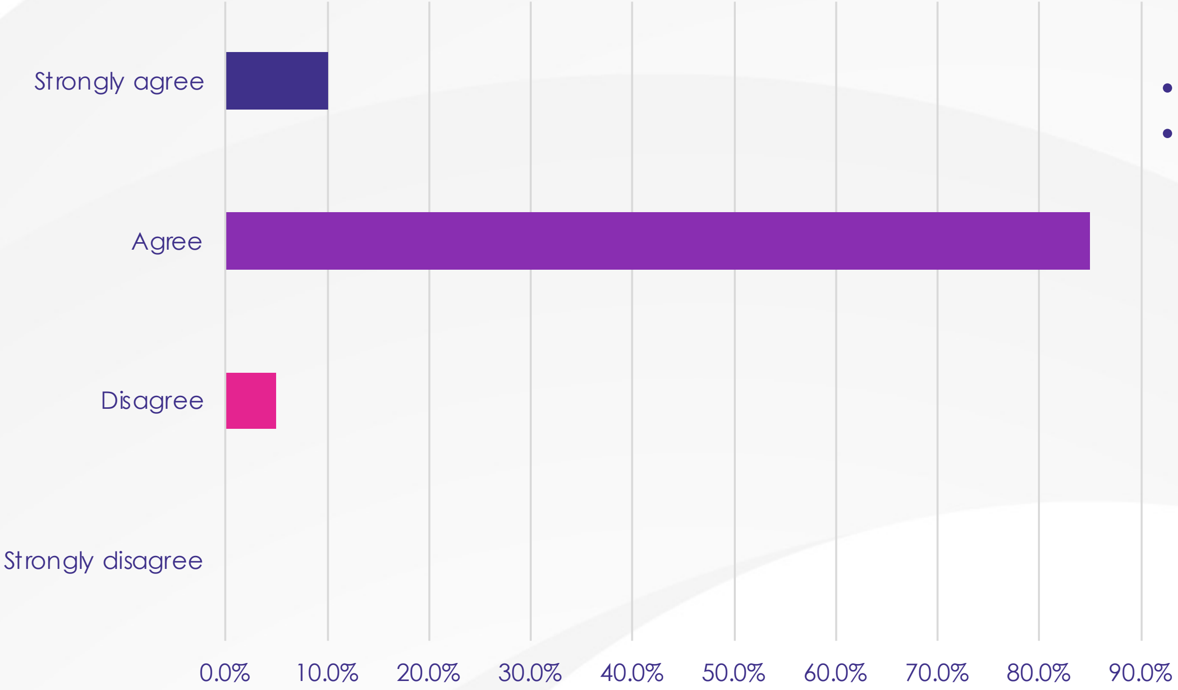

Comments

- I did not see this effect.
- It´s subjective , it´s hard to answer

Overall agreement 95%

Patients receiving treatment with ataluren seem to better manage daily situations

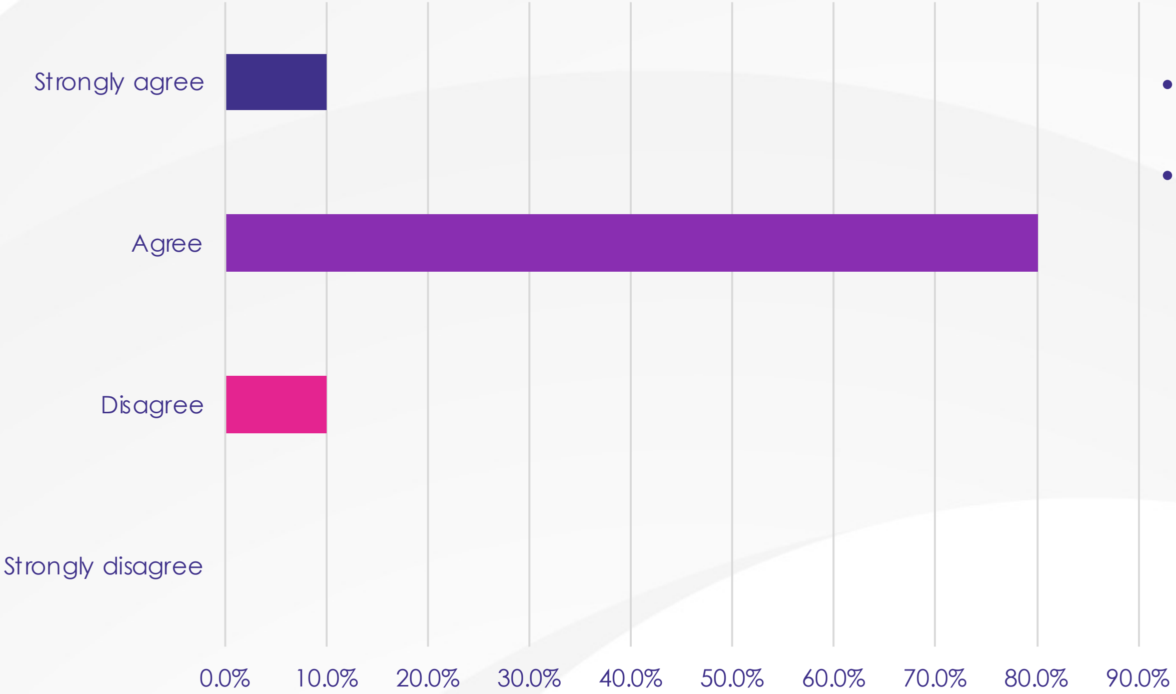

Comments

- Due to slower progression of disease it seems to be
- I am not quite sure that it improves managing daily situations beside slower progression, but I can't disagree

Overall agreement 90%

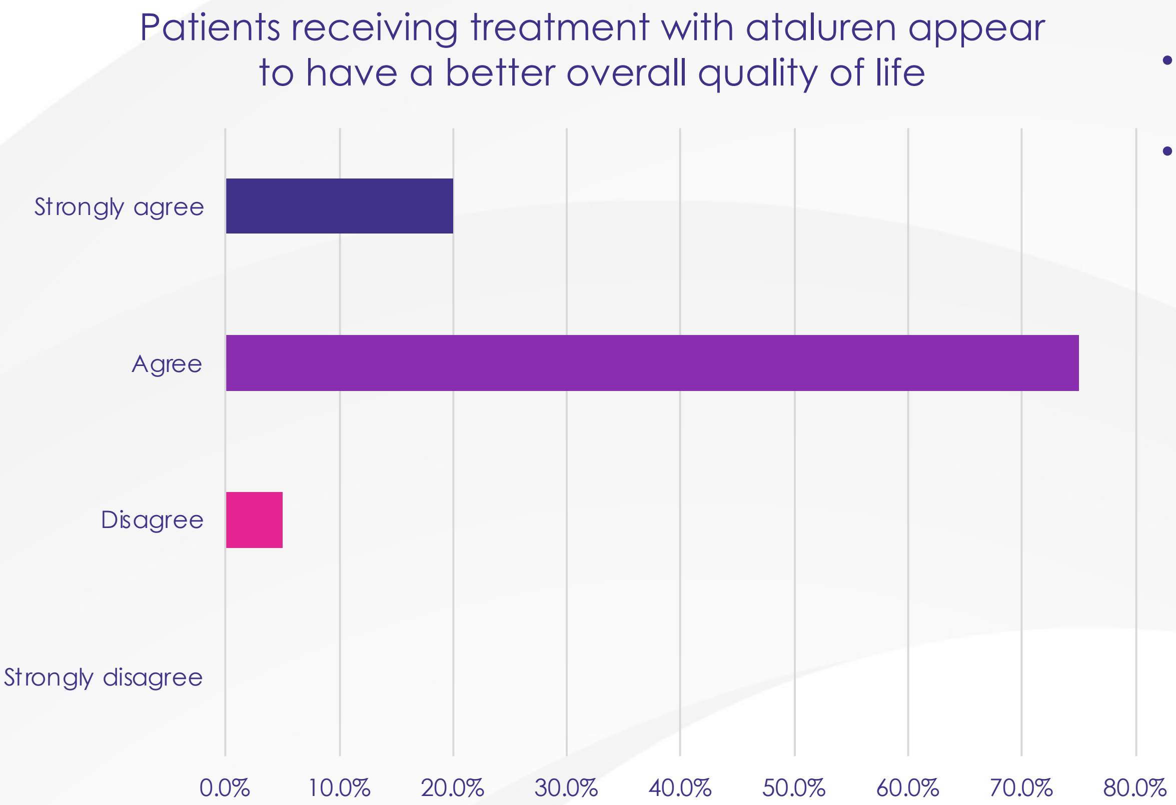

Comments

- This is the question which I'm unable to answer
- I am not sure that the patient's quality of life could be strongly influenced with any disease modifying treatment. The physician can see that the treated patient's life is different from natural history of the disease, but the patient has not got basis of comparison. He has only one life which is still strongly influenced by the disease.

Overall agreement 95%

Ataluren (in addition to standard of care) significantly delays the decline in muscle function in patients with nmDMD

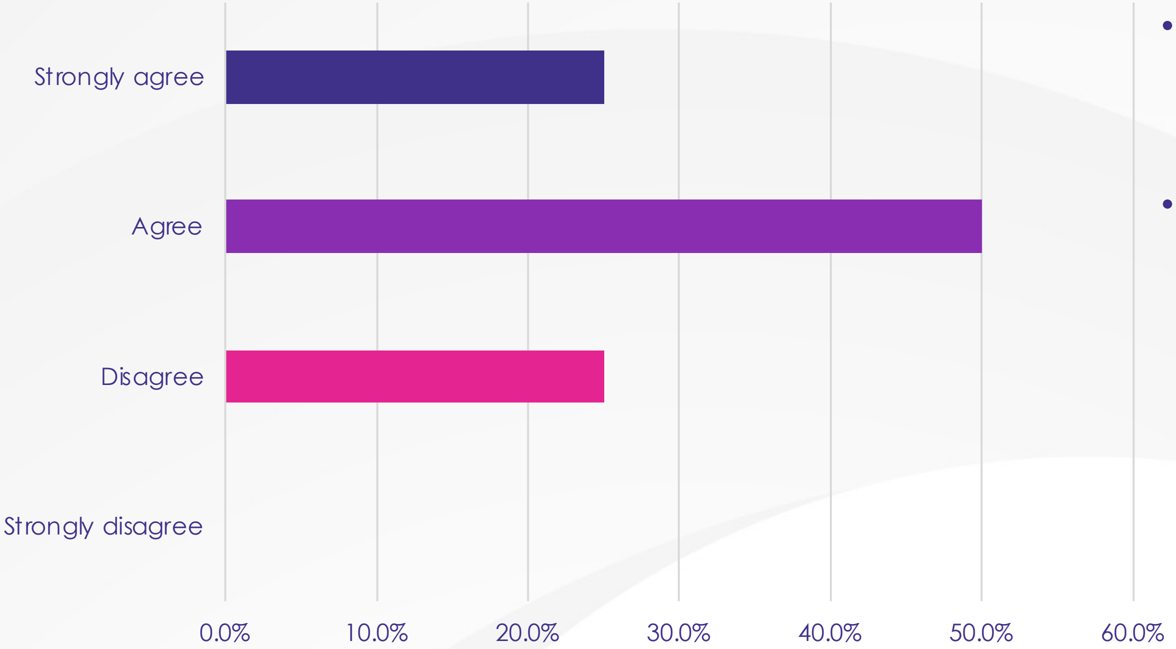

Comments

- all of our patient in ataluren older than 10 years are still ambulatory
- I still don't manage older boys with nmDMD, so can not be completely sure based on my experience
- What I saw was only mildly delayed

Overall agreement 75%

Consensus Statements
# – Loss of ambulation

Ataluren (in addition to standard of care) significantly delays the loss of ambulation in patients with nmDMD

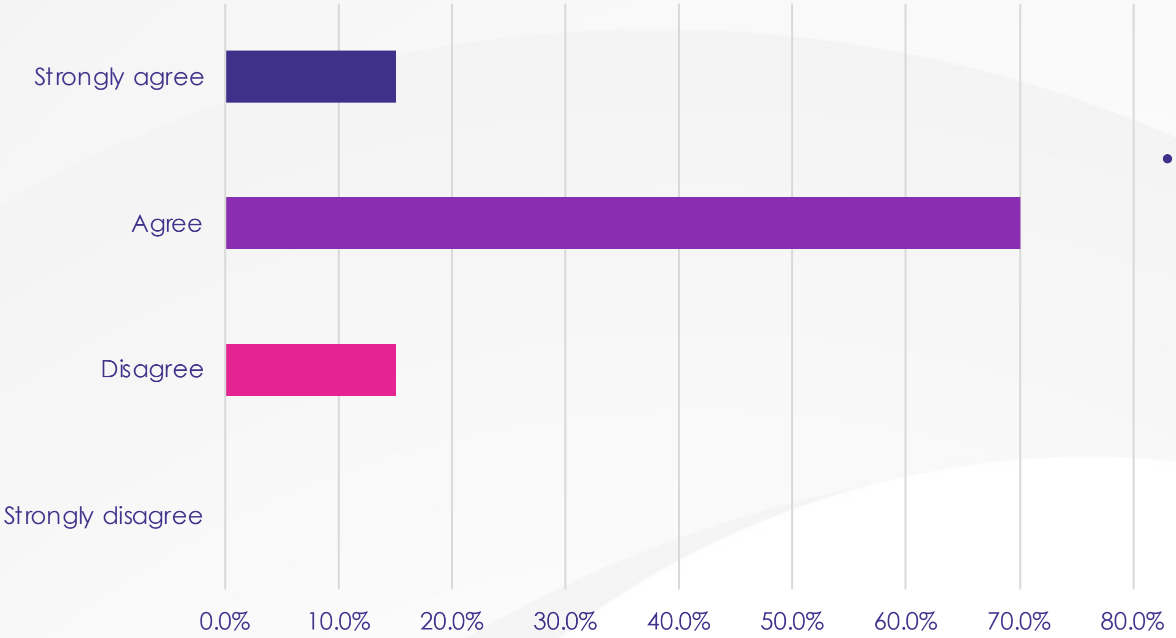

Comments

- Yes, as I already write , our oldest patient who is still ambulatory is 18 years old and all our patient over 10 years of age are still ambulatory
- It certainly delays the loss of ambulation, but not significantly

Overall agreement 85%

Ataluren (in addition to standard of care) is expected to result in the same treatment effect in each surviving muscle fibre irrespective of the nmDMD patients' ambulatory status

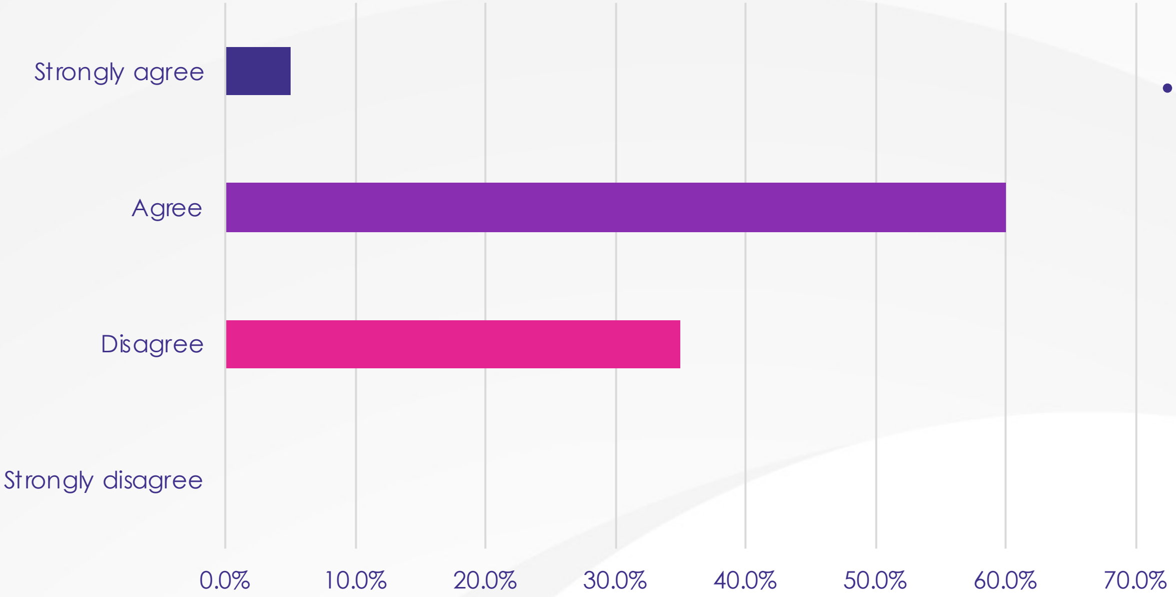

Comments

- No comments submitted

Overall agreement 65%

If a nmDMD patient receiving ataluren loses ambulation, they should continue treatment with ataluren

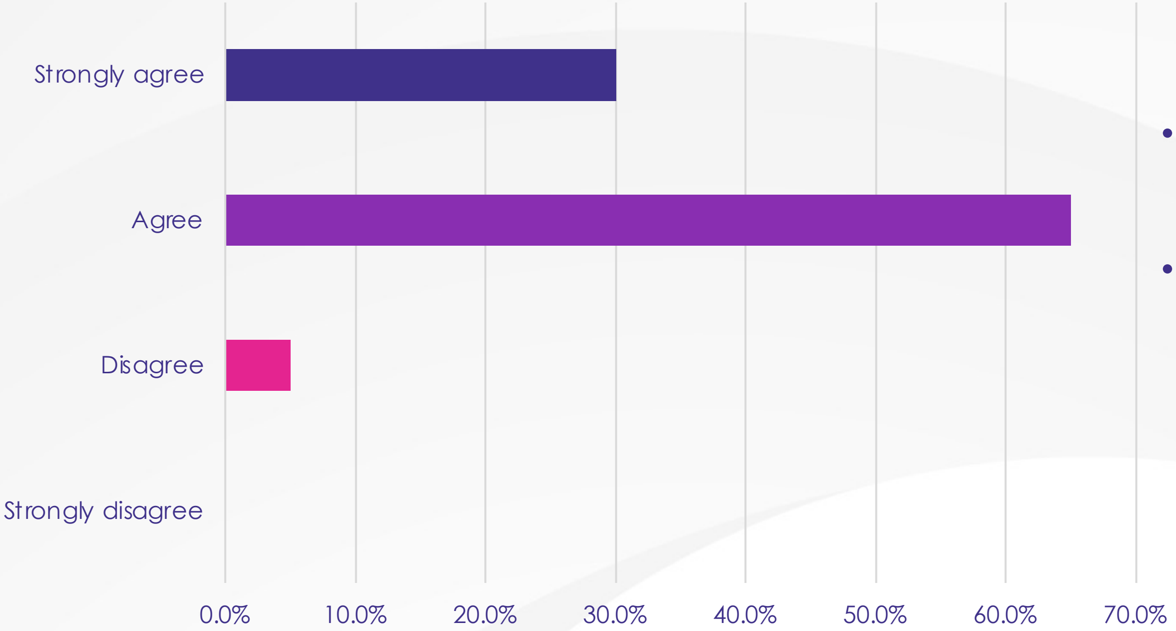

Comments

- It can slows progression of weakness in upper limbs, in respiratory muscles..
- They should but actually it is not supported

Overall agreement 95%

There is life beyond loss of ambulation. There are still lots of important functions of the muscles, such as being able to use the hands and arms, fine motor skills and respiratory muscles – all these functions should be maintained for as long as possible

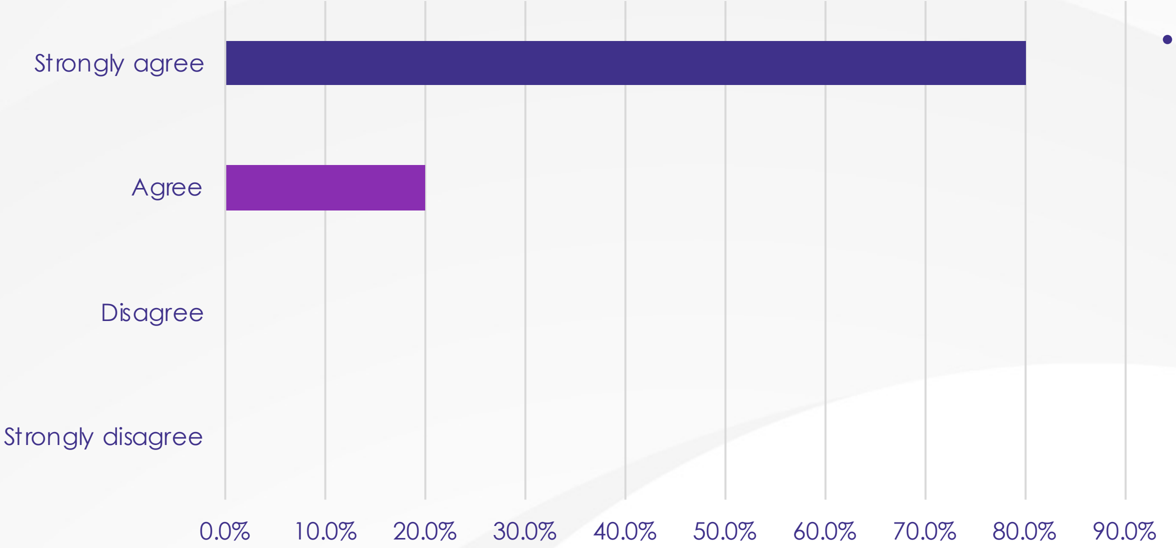

Comments

- No comments submitted

Overall agreement 100%

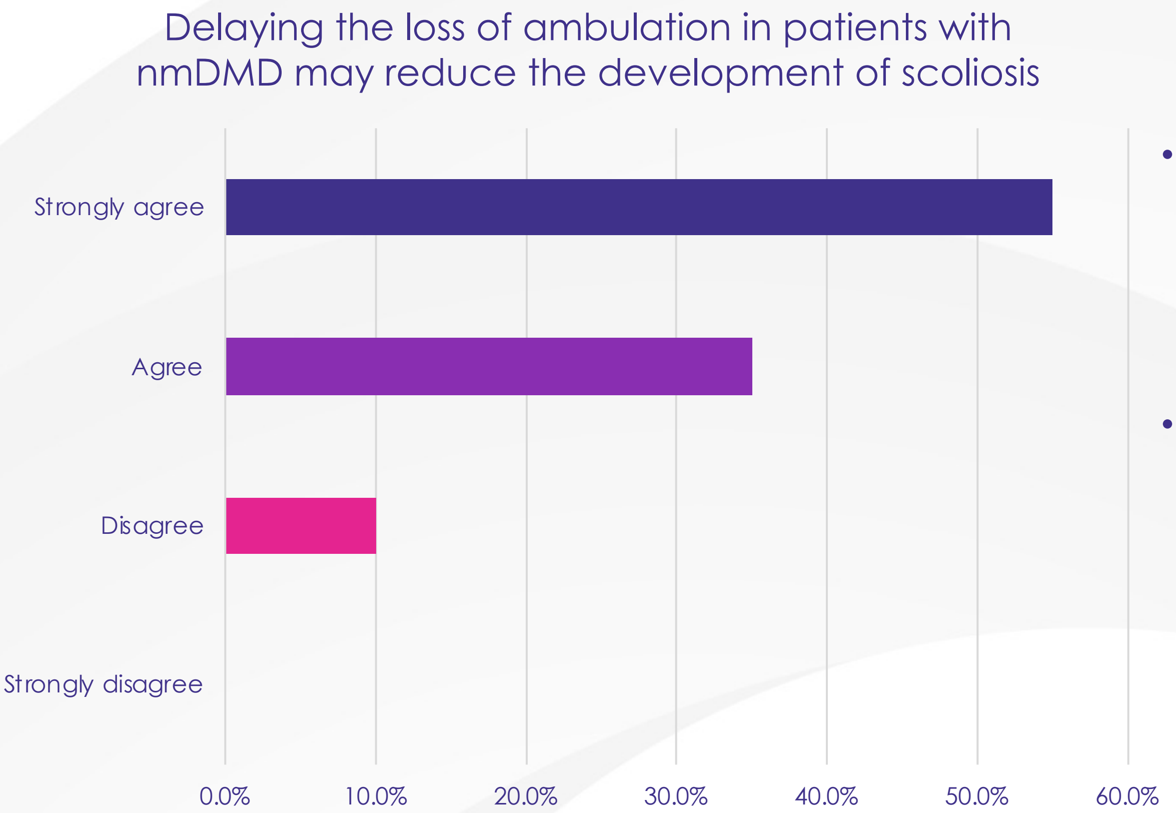

Comments

- If it delayed progression of muscle weakness in lower limb it slows progression in all muscles group also core muscles an important is ability to stand
- Loss of ambulation in addition with disease progression and adolescence deteriorate scoliosis

Overall agreement 90%

Delaying the loss of ambulation in patients with nmDMD delays the decline of respiratory function

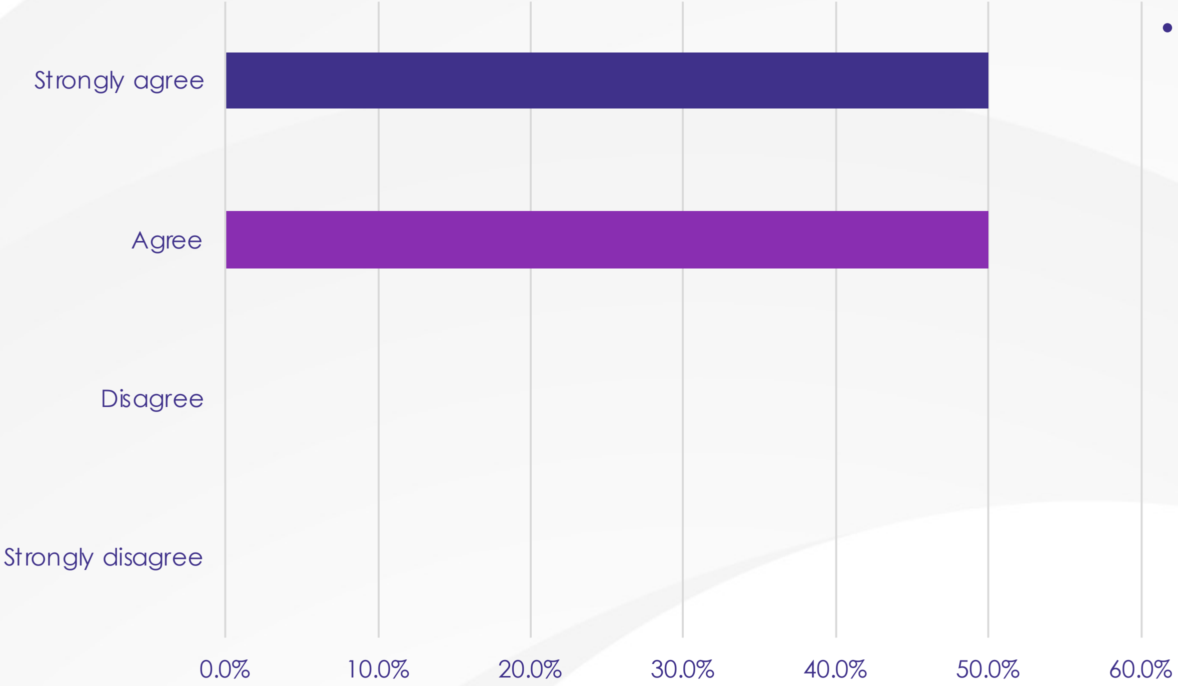

Comments

- it similar than answer before it slows also progression of respiratory muscles

Overall agreement 100%

Delaying the loss of ambulation in patients with nmDMD is related to the decline of upper limb function

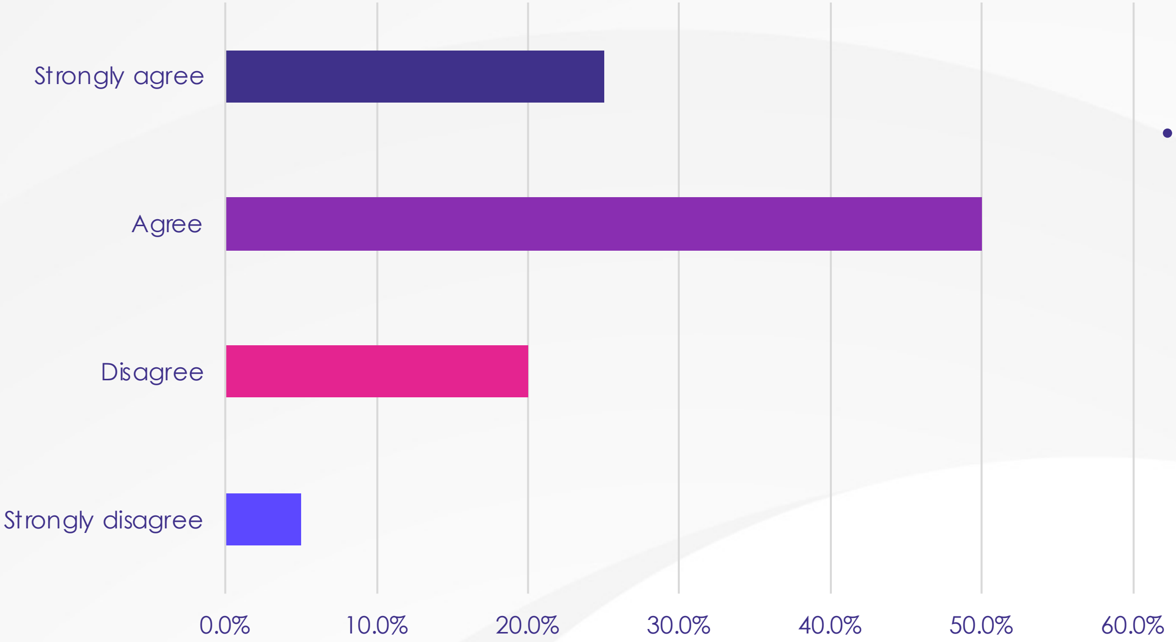

Comments

- No comments submitted

Overall agreement 75%

Consensus Statements – Scoliosis

Development of scoliosis has a detrimental impact on patients' pulmonary function

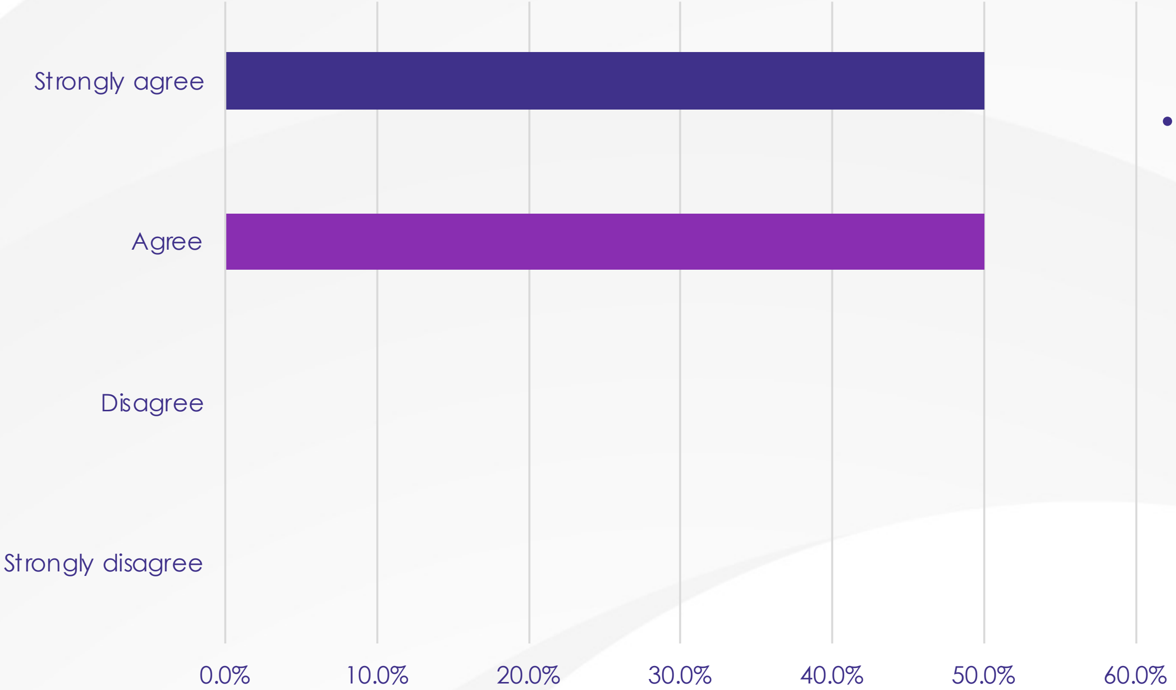

Comments

- It change parameters in thorax and its associated with core muscles weakness and respiratory muscle weakness so it affects pulmonary function in our patient

Overall agreement 100%

nmDMD patients treated with ataluren (in addition to standard of care) are less likely to develop scoliosis

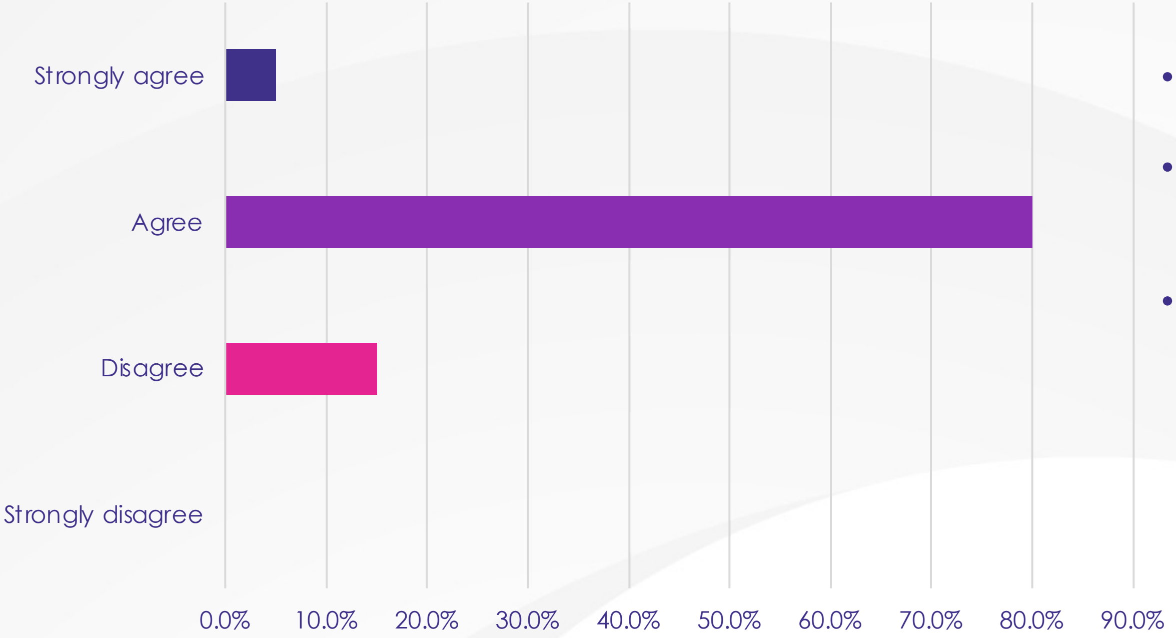

Comments

- I don t have a clear response here
- Our are patient are still ambulatory and has minimal scoliosis
- Do not know

Overall agreement 85%

Non-ambulatory nmDMD patients are less likely to develop scoliosis if they continue treatment with ataluren (in addition to standard of care) after loss of ambulation

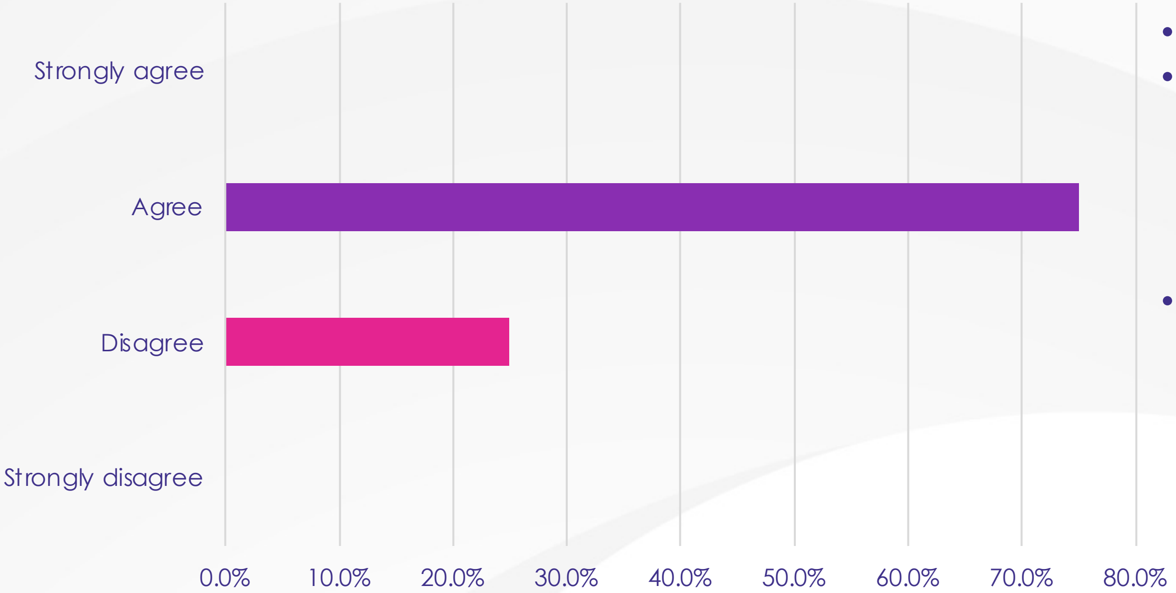

Comments

- Not sure
- we don´t have any non-ambulatory patient on treatment so i don´t have experience with it but my expectation is that yes
- no data I don't know

Overall agreement 75%

Consensus Statements – Upper limb function

Delaying the decline of muscle function in patients' upper limbs helps to maintain independence

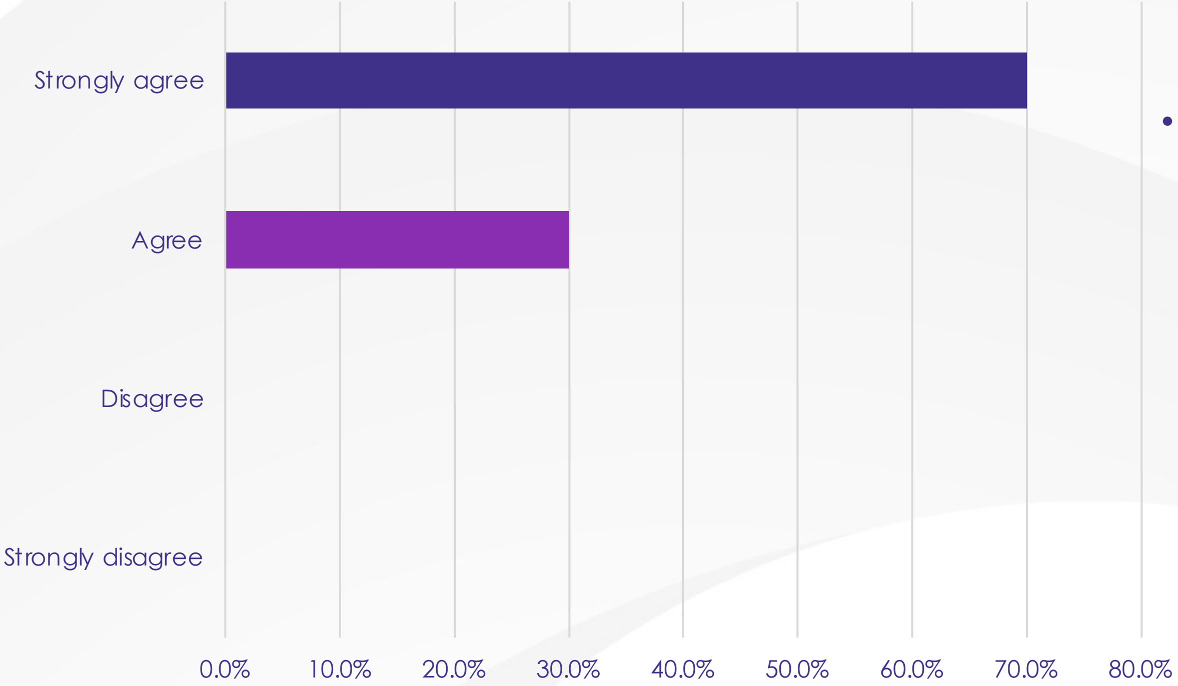

Comments

- No comments submitted

Overall agreement 100%

Delaying in the decline in fine motor skills also enables patients in wheelchairs to continue to be as independent as possible

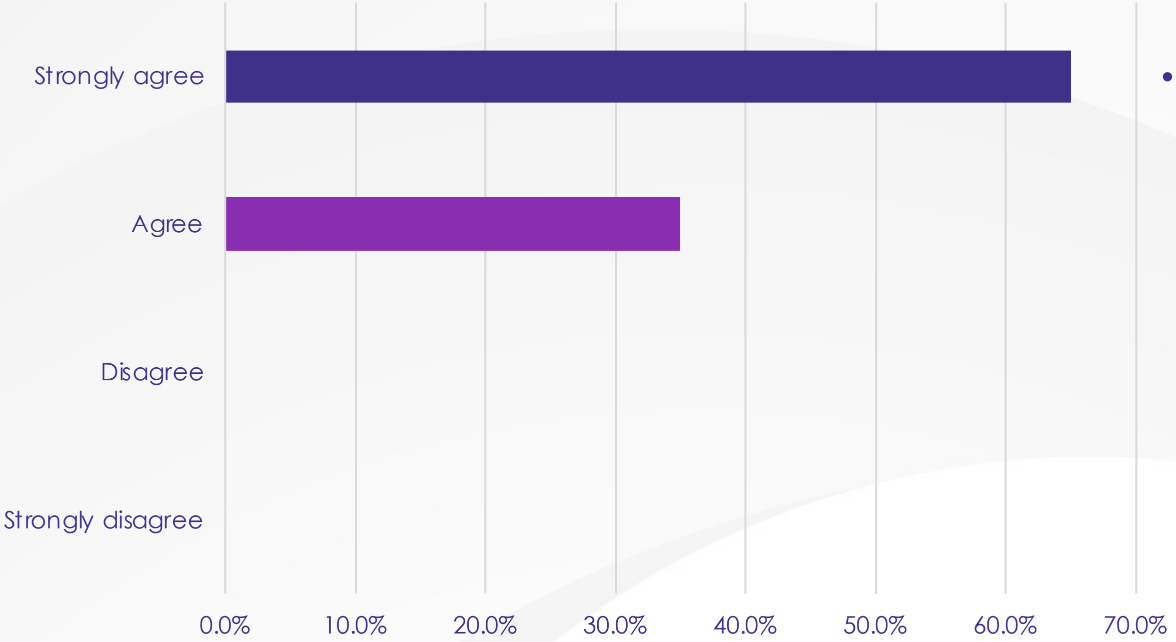

Comments

- No comments submitted

Overall agreement 100%

Delaying the decline of upper limb strength enables non-ambulatory patients to transfer from their wheelchair to the toilet, maintain intimate hygiene, retain independence and protect their quality of life

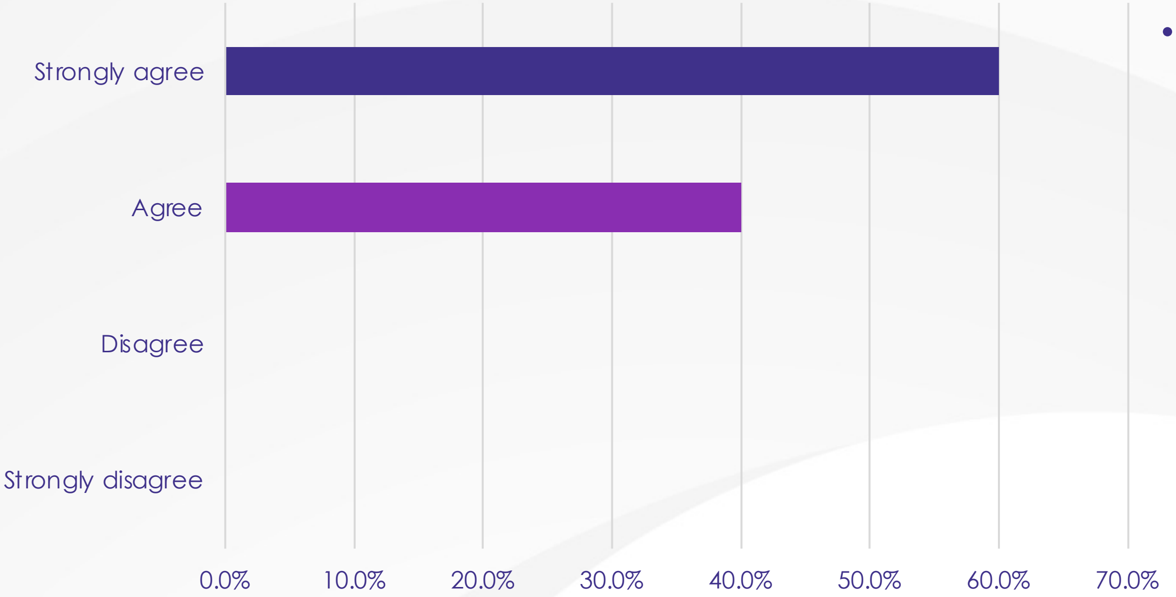

Comments

- No comments submitted

Overall agreement 100%

Decline of upper limb function has a major impact on patients’ quality of life; they become increasingly dependant on others

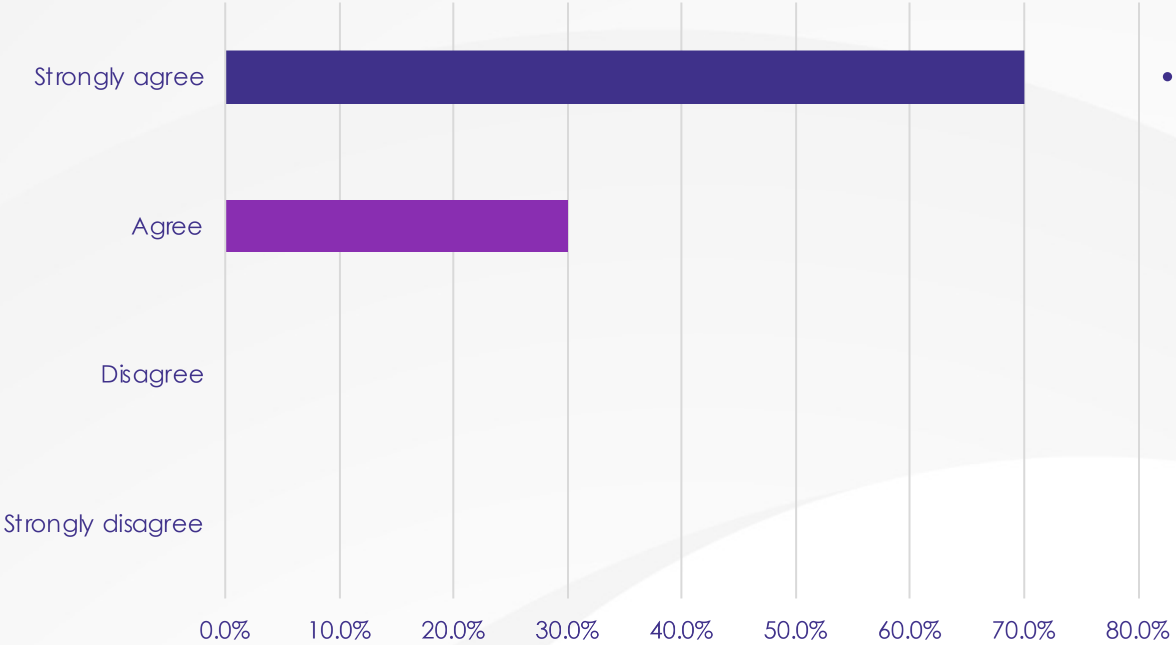

Comments

- No comments submitted

Overall agreement 100%

Ataluren (in addition to standard of care) delays the decline in nmDMD patients' upper limb function, regardless of mobility status

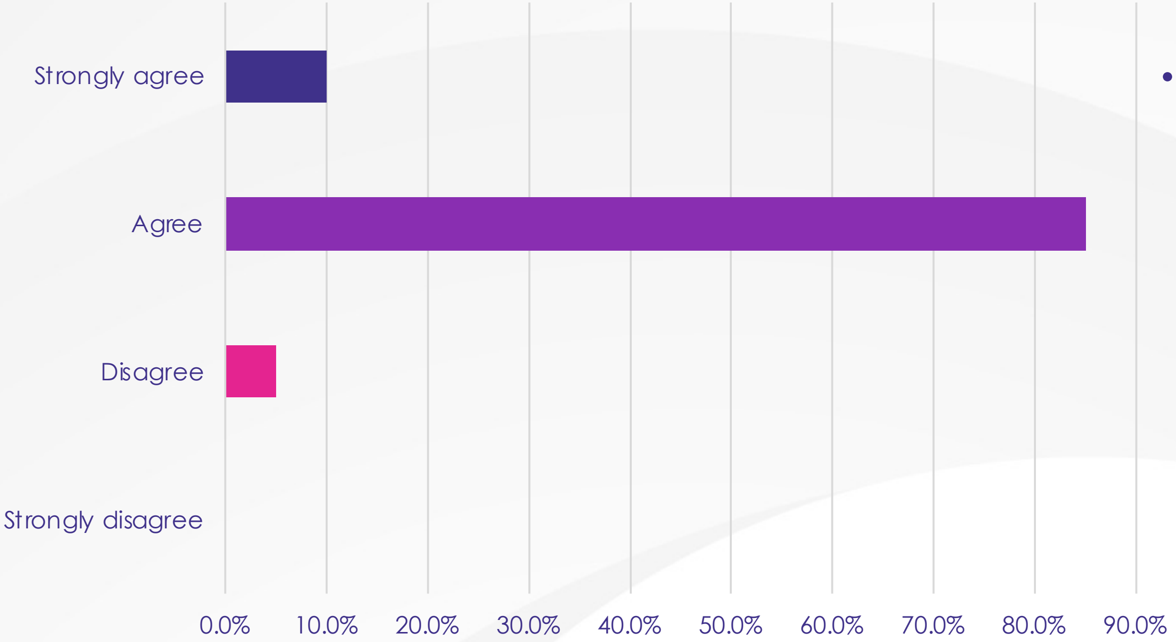

Comments

- we don´t have this experience with non ambulatory patient but my expectation are yes

Overall agreement 95%

Ataluren (in addition to standard of care) delays the decline of fine motor skills in nmDMD patients

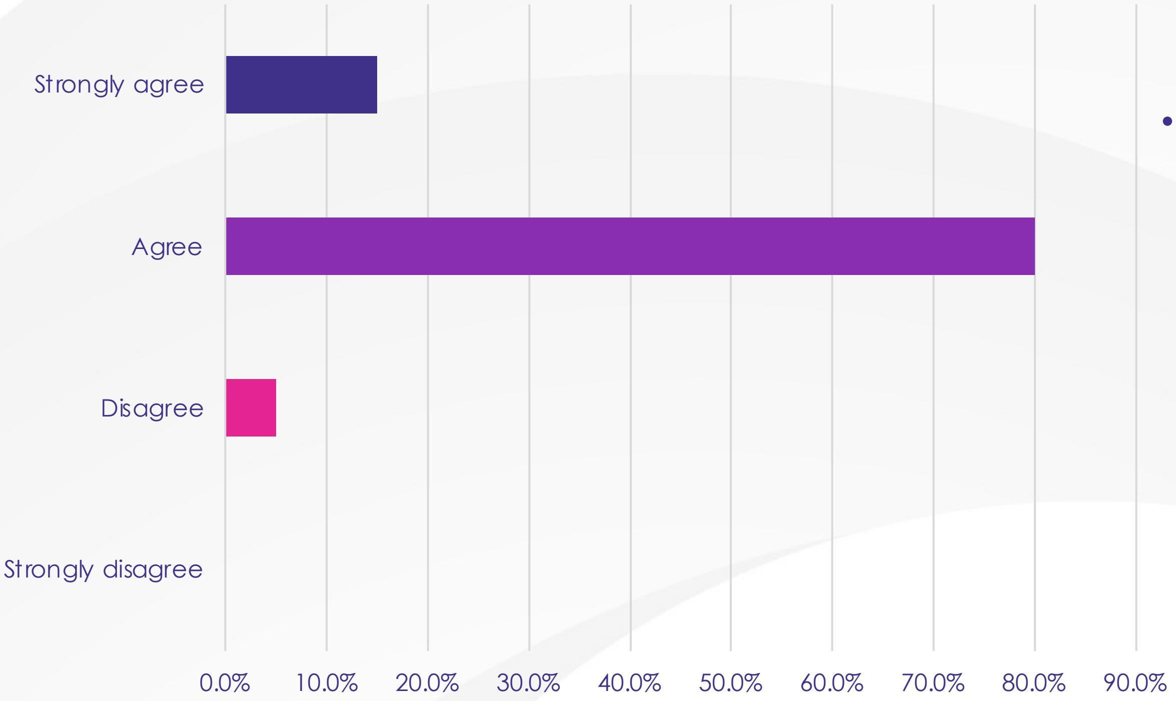

Comments

- it delays the decline of upper limbs muscle strength so it helps to preserve fine motor skills

Overall agreement 95%

Consensus Statements – Pulmonary function

Continuing the use of ataluren, in addition to standard of care, in nmDMD patients when they lose ambulation delays the decline in pulmonary function

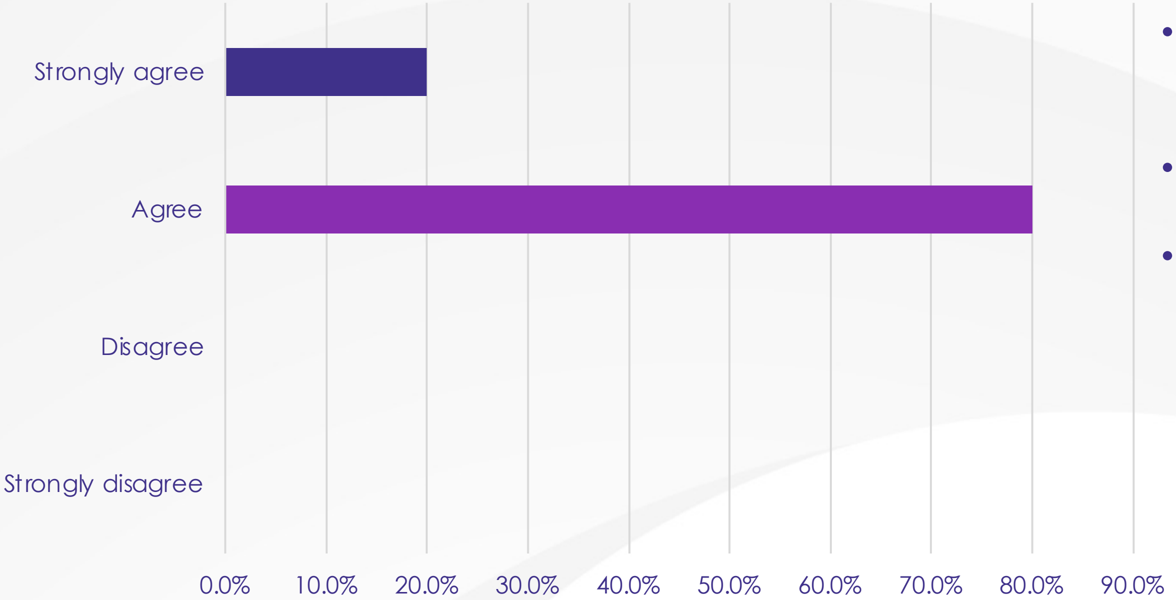

Comments

- if there is effect on all muscles group than also on respiratory muscles
- I do not have my own experience in this.
- Possibly

Overall agreement 100%

Maintaining patients' pulmonary function means they experience fewer respiratory infections and may require less frequent hospitalisations

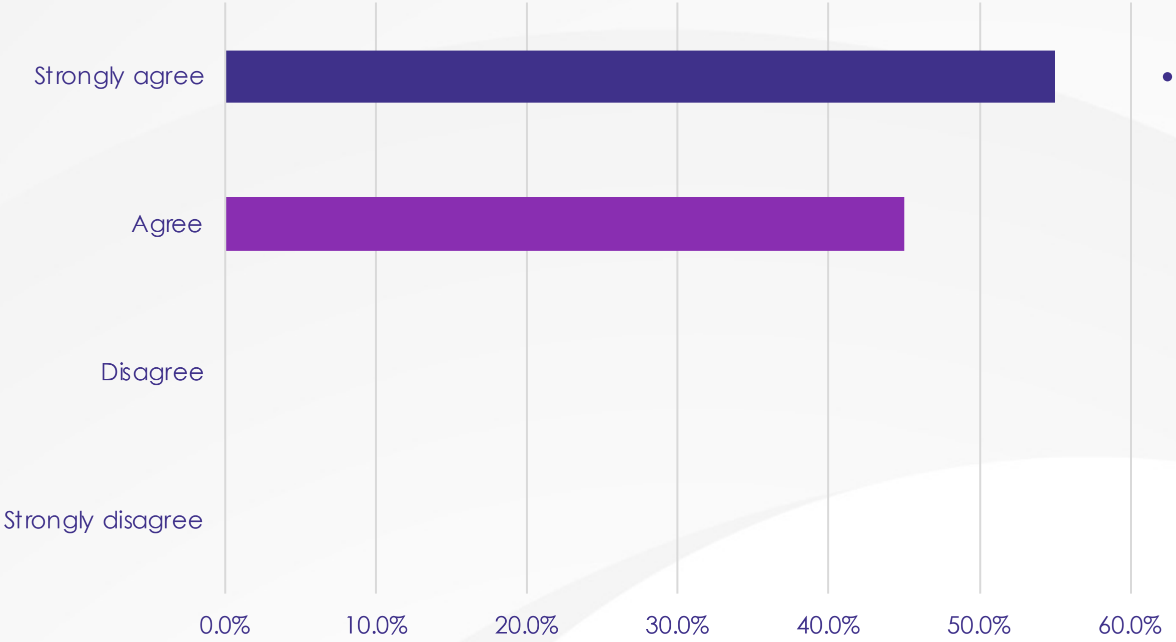

Comments

- No comments submitted

Overall agreement 100%

The ability of nmDMD patients to cough is maintained for longer with ataluren, in addition to standard of care

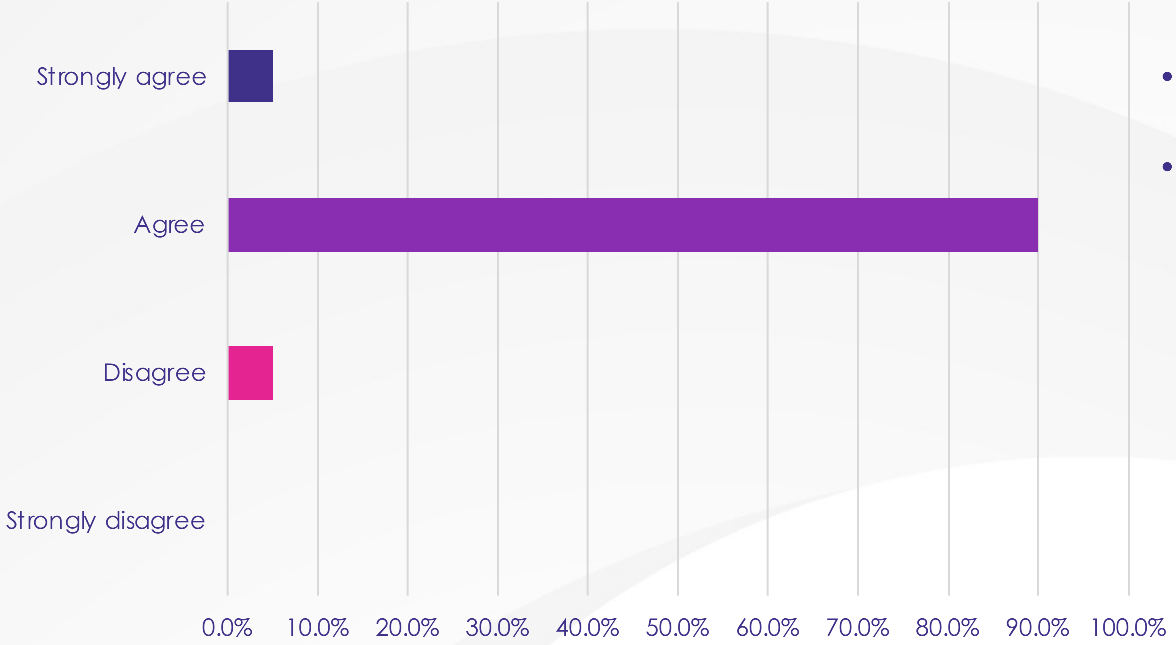

Comments

- All our patient on ataluren are able to cough
- Possibly

Overall agreement 95%

Ataluren, in addition to standard of care, delays the decline in nmDMD patients' pulmonary function

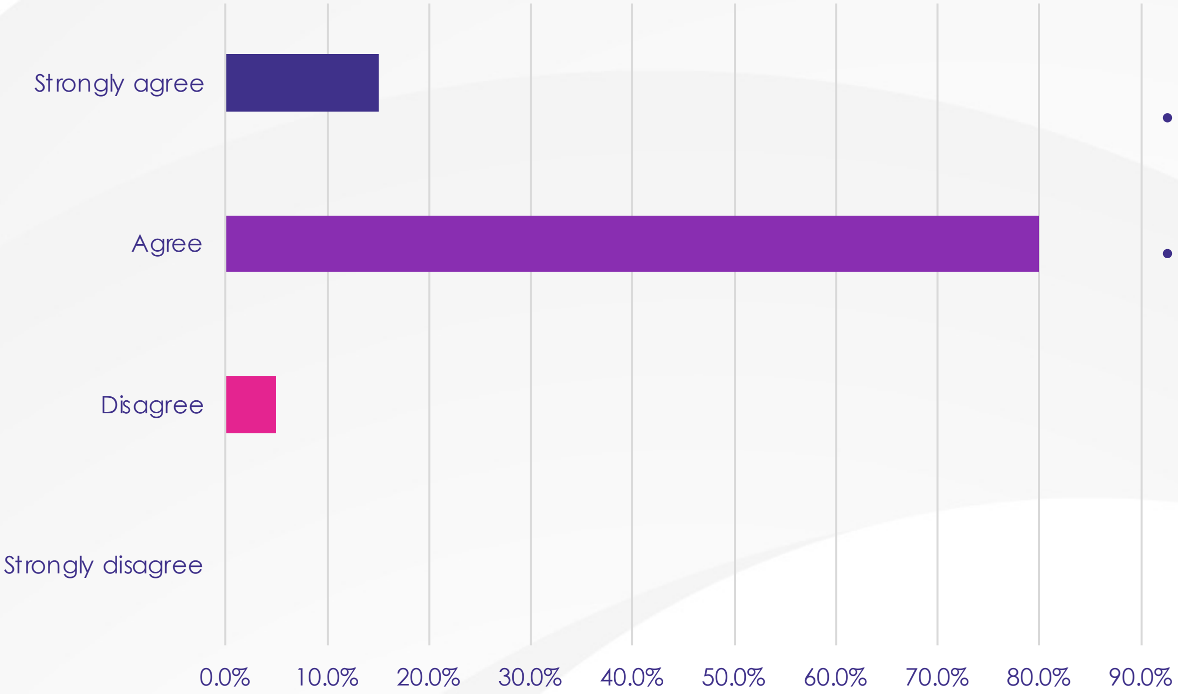

Comments

- any of our patient on ataluren treatment need ventilatory support
- Same as 34

Overall agreement 95%

Ataluren, in addition to standard of care, significantly delays the decline in nmDMD patients' pulmonary function

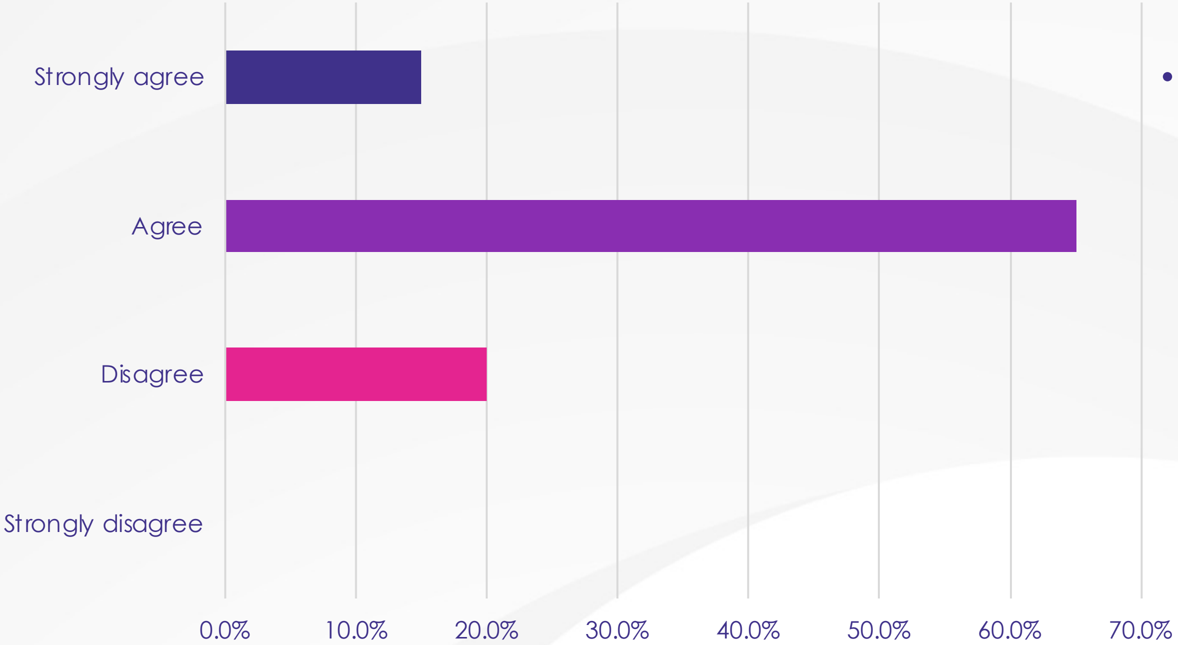

Comments

- I am not sure about the significantly term...

Overall agreement 80%

Ataluren, in addition to standard of care, prolongs nmDMD patients' ability to breathe independently

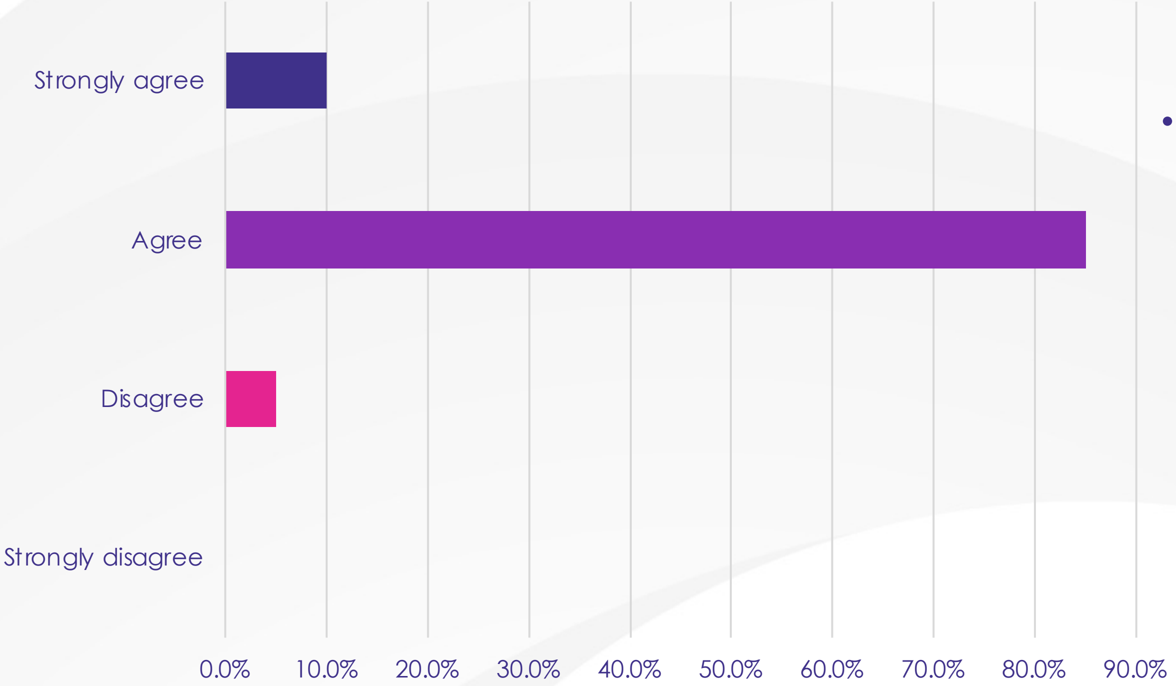

Comments

- No comments submitted

Overall agreement 95%

Ataluren, in addition to standard of care, significantly prolongs nmDMD patients' ability to breathe independently

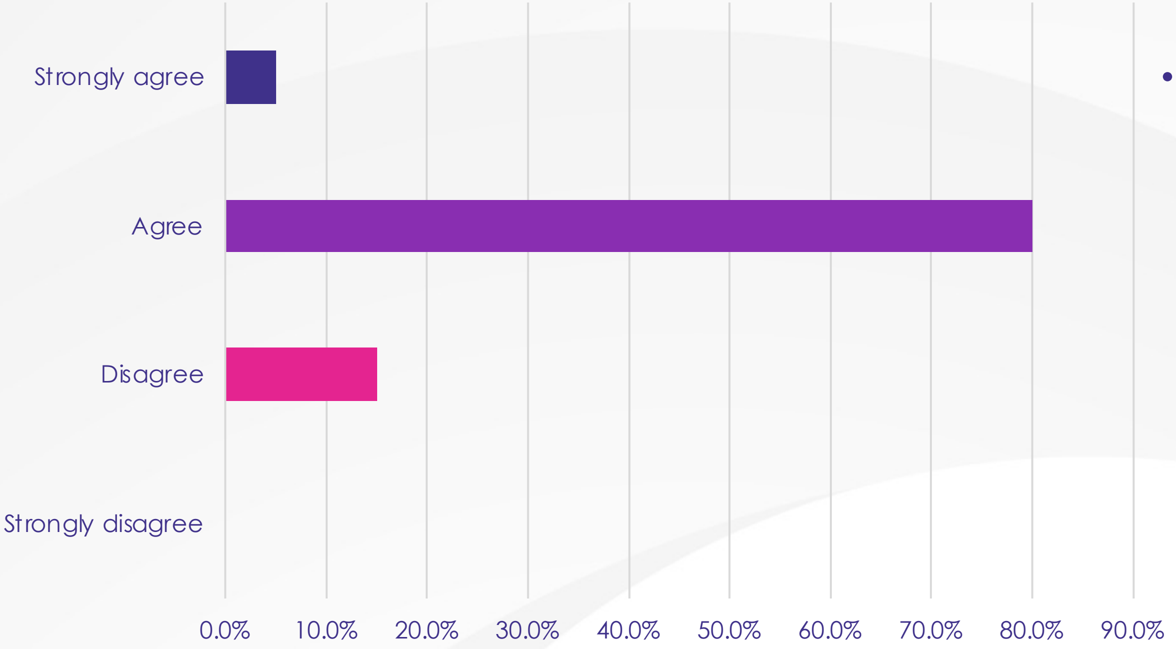

Comments

- No comments submitted

Overall agreement 85%

When nmDMD patients' FVC falls below 60%, at latest, it becomes necessary to commence physiotherapy and/or screening for night-time ventilation

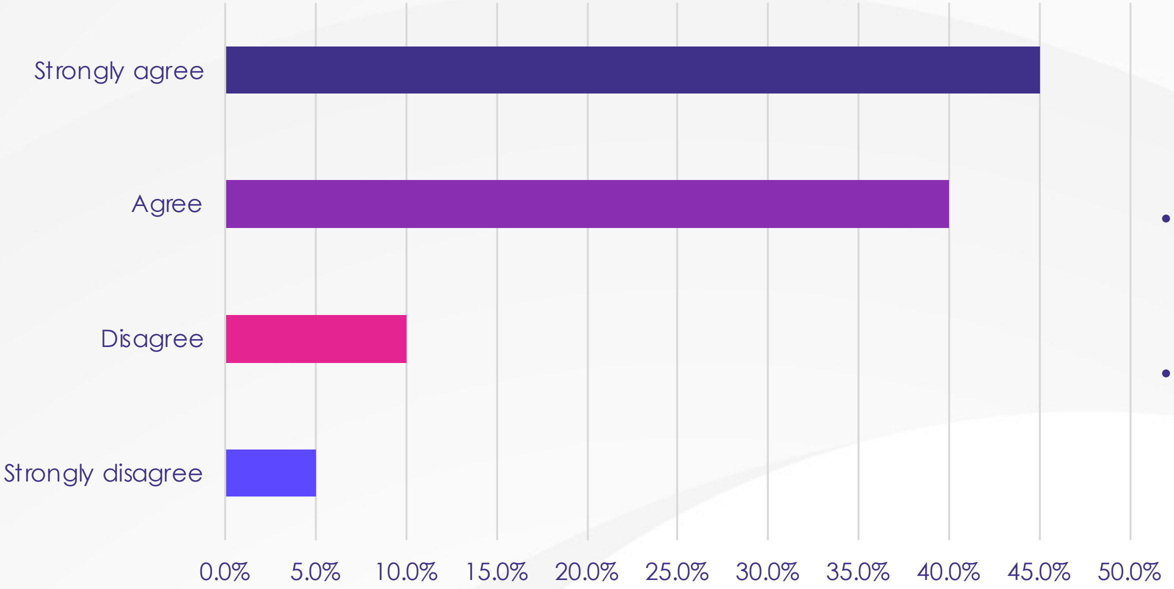

Comments

- we perform it in our patient
- Even earlier, there are also other factors that we monitor to screen patients that start to show ventilatory problems. Regular respiratory physiotherapy also maintains good pulmonary function and prevents hospitalisations due to respiratory infections.
- I think physiotherapy and/or screening for night-time ventilation should be performed regularly from the time of diagnosis of DMD
- physiotherapy should be started as soon as the diagnosis is made and screening for night-time ventilation too

Overall agreement 85%

Patients that continue to receive ataluren, in addition to standard of care, after loss of ambulation are expected to have a delayed requirement for ventilation

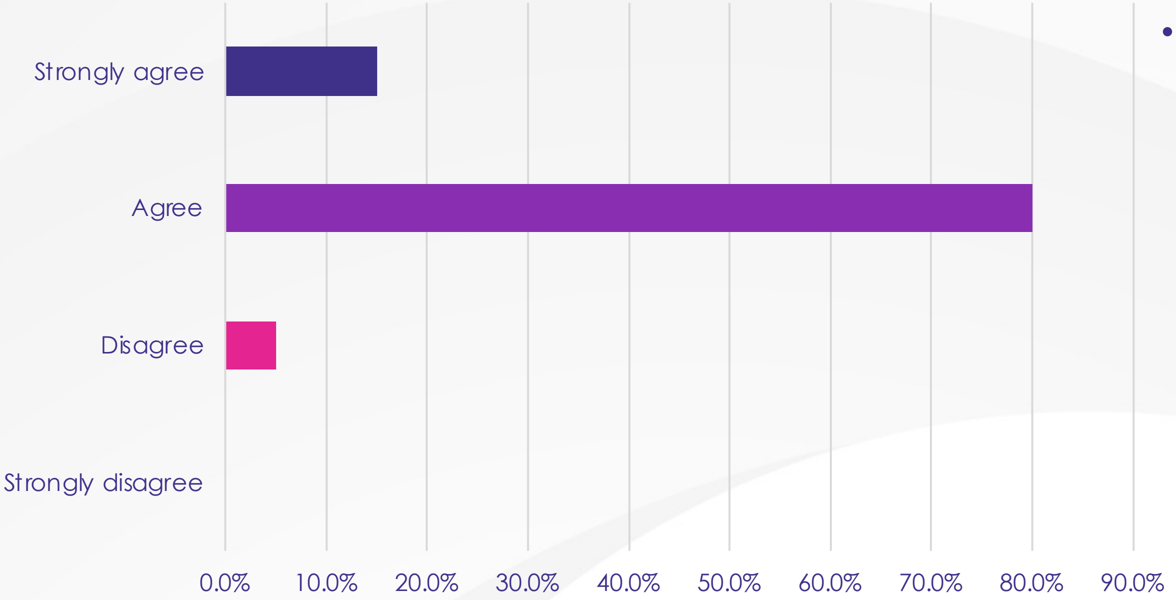

Comments

- No comments submitted

Overall agreement 95%

Consensus Statements – Cardiac function

It is logical to expect ataluren in nmDMD patients to have an effect on all muscles, including the cardiac muscle

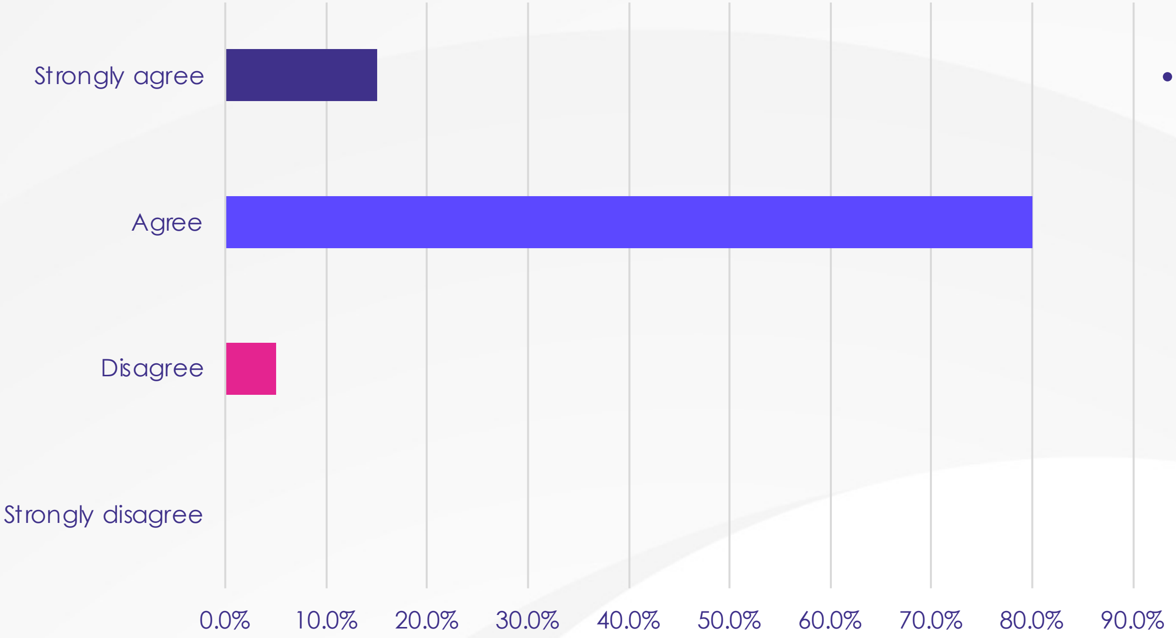

Comments

- I don't know

Overall agreement 95%

It is logical to expect Ataluren, in addition to standard of care, to delay the onset of cardiac decline in patients with nmDMD

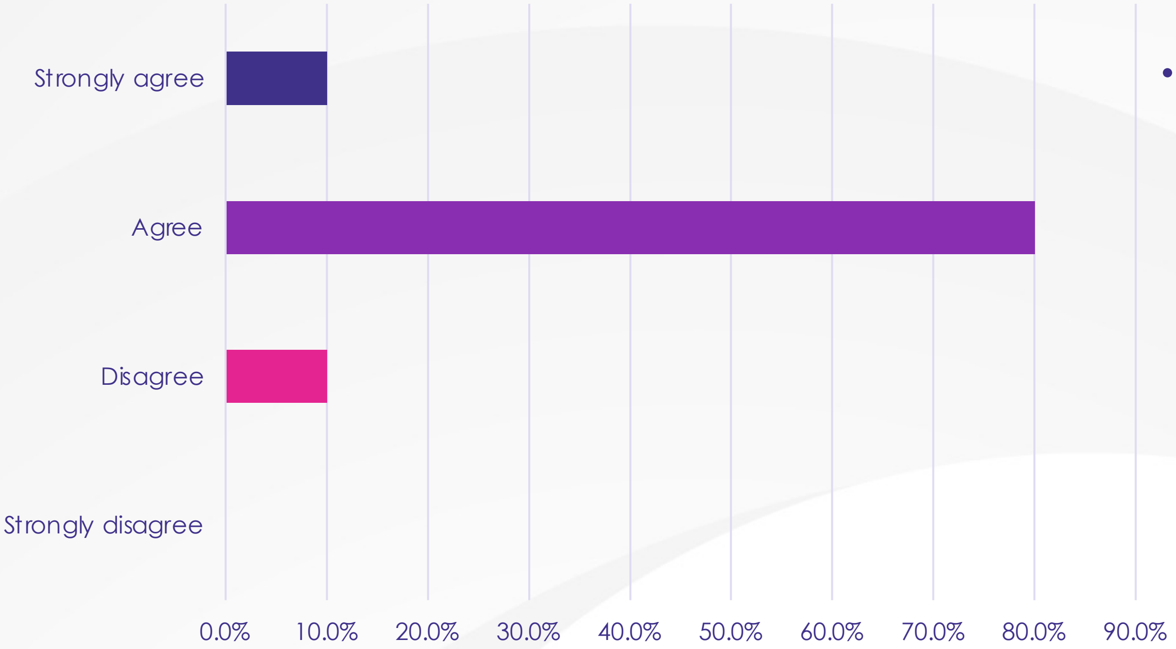

Comments

- No comments submitted

Overall agreement 90%

It is logical to expect Ataluren, in addition to standard of care, to delay the onset of cardiomyopathy in patients with nmDMD

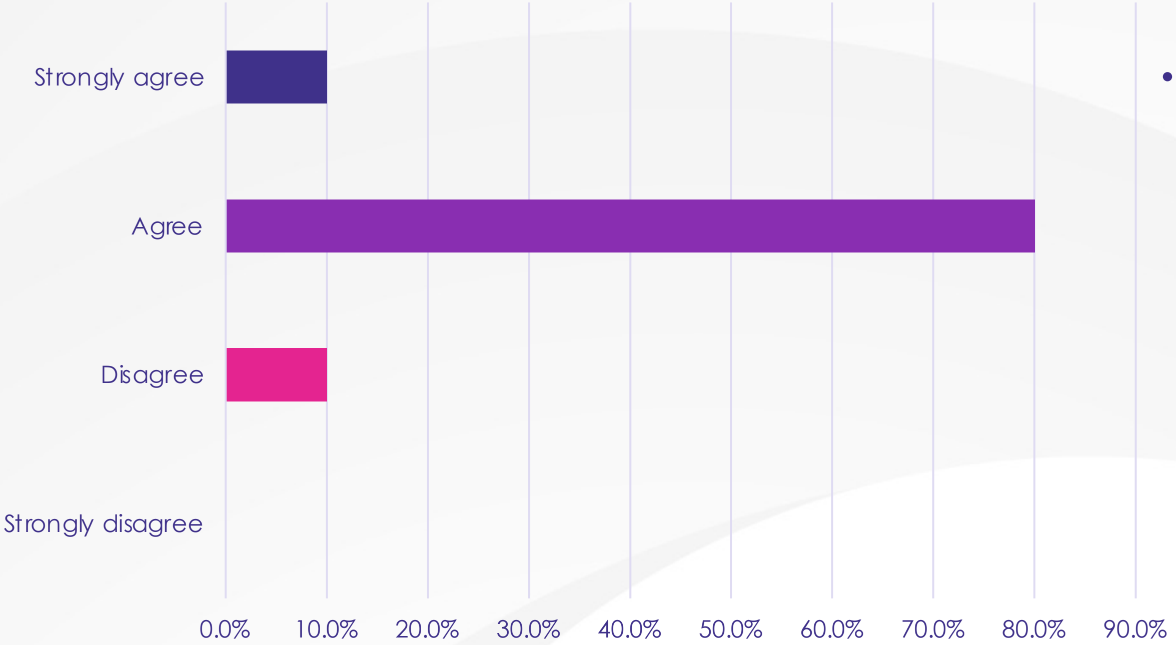

Comments

- No comments submitted

Overall agreement 90%

It is logical to expect Ataluren, in addition to standard of care, to delay the decline in left ventricular ejection fraction in patients with nmDMD

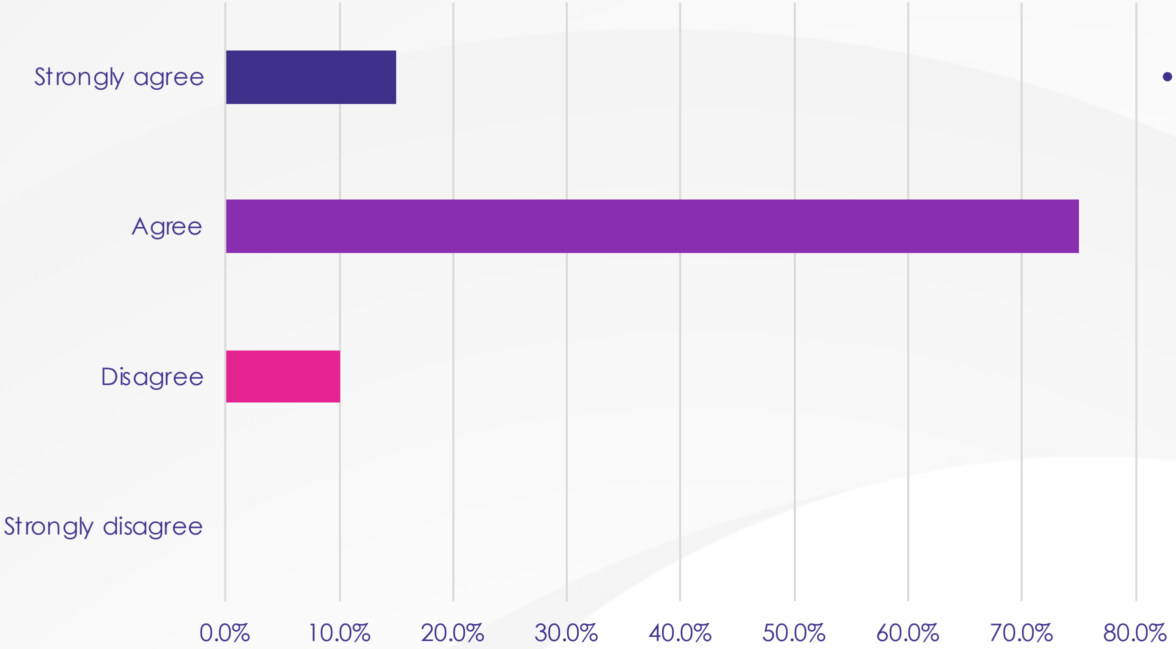

Comments

- To all above in this section, it is logical to carefully watch cardiac function as it can be affected with ataluren treatment

Overall agreement 90%

Consensus Statements
# – Duration of treatment with ataluren

It is important to preserve the function of even small muscles in nmDMD patients

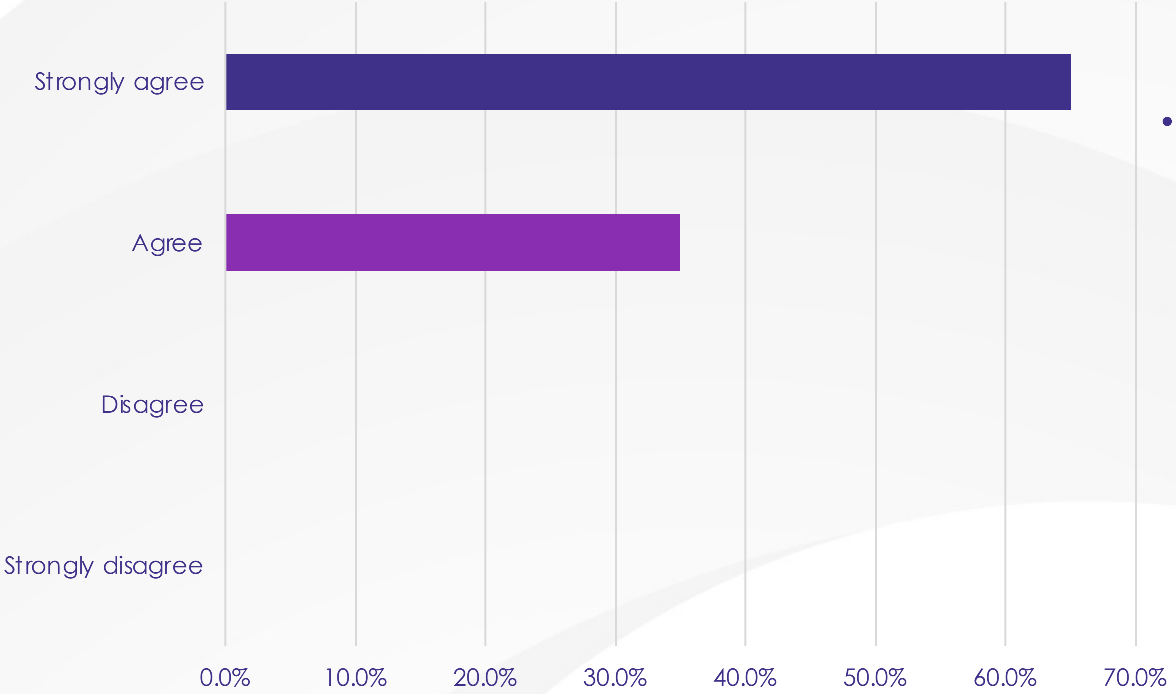

Comments

- No comments submitted

Overall agreement 100%

Treatment with ataluren should be continued as long as both the physician and nmDMD patient are both willing to continue treatment

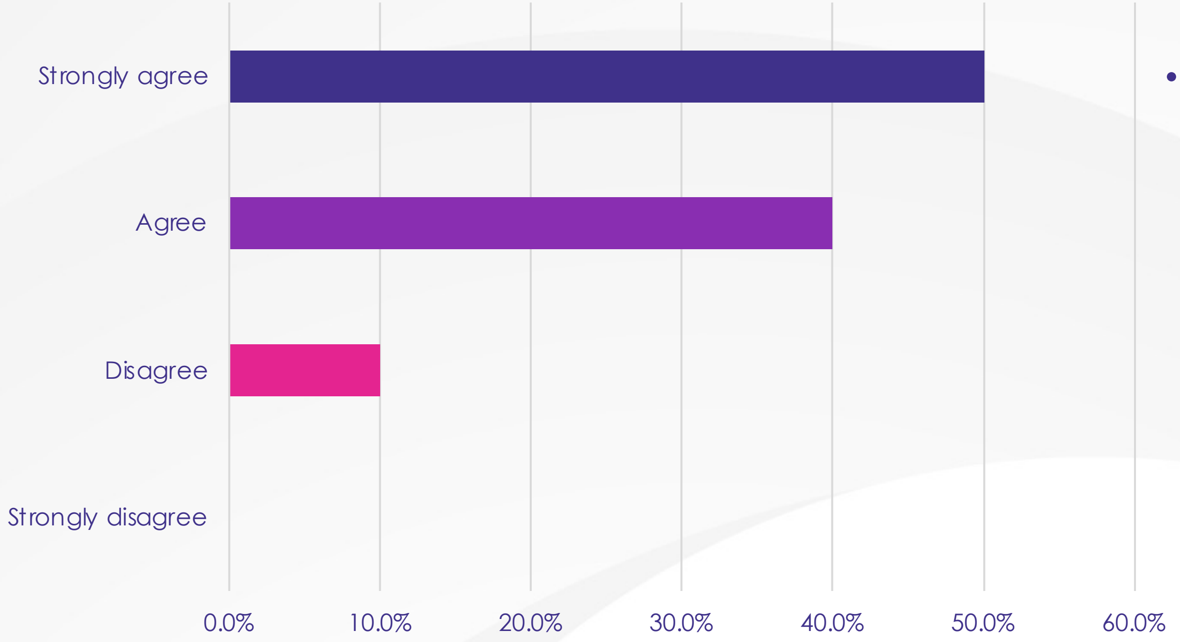

Comments

- As long as the treatment is effective (in non ambulatory patients)

Overall agreement 90%

As long as nmDMD patients still have functionally important muscles that can be influenced by ataluren they should continue to be treated

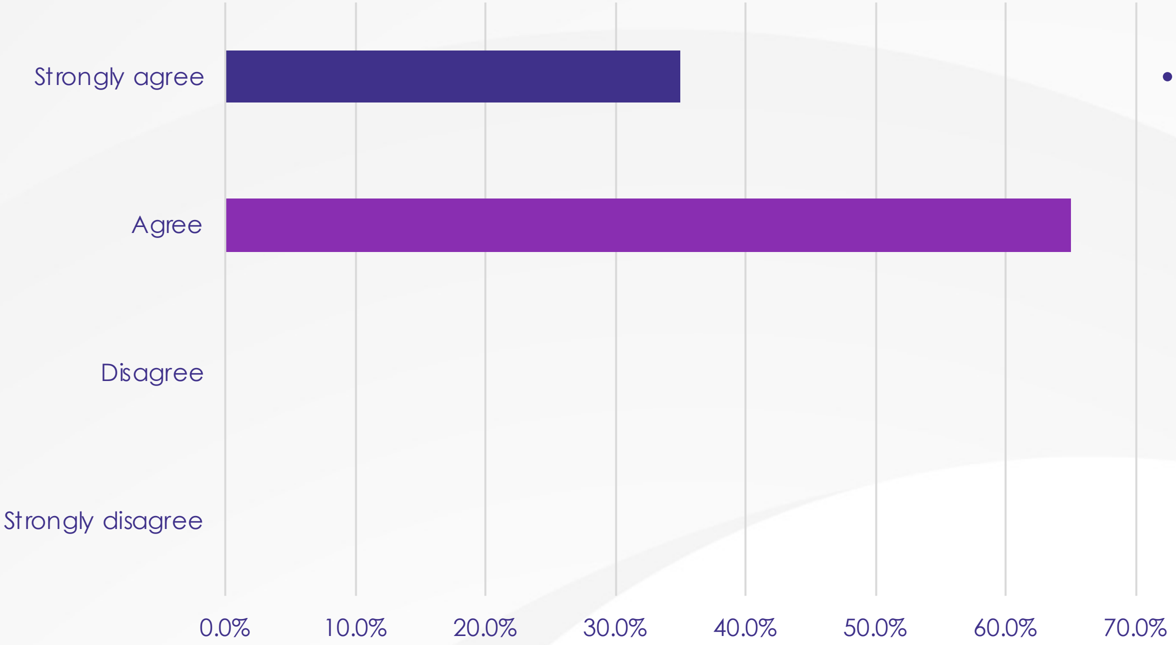

Comments

- No comments submitted

Overall agreement 100%
